# Signature laminar distributions of pathology in frontotemporal lobar degeneration

**DOI:** 10.1007/s00401-021-02402-3

**Published:** 2022-01-08

**Authors:** Daniel T. Ohm, Katheryn A. Q. Cousins, Sharon X. Xie, Claire Peterson, Corey T. McMillan, Lauren Massimo, Katya Raskovsky, David A. Wolk, Vivianna M. Van Deerlin, Lauren Elman, Meredith Spindler, Andres Deik, John Q. Trojanowski, Edward B. Lee, Murray Grossman, David J. Irwin

**Affiliations:** 1 Penn Digital Neuropathology Lab, Department of Neurology, Hospital of the University of Pennsylvania, University of Pennsylvania Perelman School of Medicine, 3600 Spruce Street, Philadelphia, PA 19104 USA; 2 Penn Frontotemporal Degeneration Center, Department of Neurology, Perelman School of Medicine, University of Pennsylvania, Philadelphia, PA 19104 USA; 3 Department of Biostatistics, Epidemiology, and Informatics, Perelman School of Medicine, University of Pennsylvania, Philadelphia, PA 19104 USA; 4 Penn Alzheimer’s Disease Research Center, Department of Neurology, Perelman School of Medicine, University of Pennsylvania, Philadelphia, PA 19104 USA; 5 Penn Memory Center, Department of Neurology, Perelman School of Medicine, University of Pennsylvania, Philadelphia, PA 19104 USA; 6 Department of Pathology and Laboratory Medicine, Perelman School of Medicine, University of Pennsylvania, Philadelphia, PA 19104 USA; 7 Comprehensive Amyotrophic Lateral Sclerosis Center,Department of Neurology, Perelman School of Medicine, University of Pennsylvania, Philadelphia, PA 19104 USA; 8 Parkinson’s Disease and Movement Disorders Center, Department of Neurology, Perelman School of Medicine, University of Pennsylvania, Philadelphia, PA 19107 USA; 9 Center for Neurodegenerative Disease Research, Department of Pathology and Laboratory Medicine, Perelman School of Medicine, University of Pennsylvania, Philadelphia, PA 19104 USA

**Keywords:** Frontotemporal lobar degeneration, Tau, TDP-43, Laminar pathology, Supragranular, Infragranular

## Abstract

Frontotemporal lobar degeneration (FTLD) with either tau (FTLD-tau) or TDP-43 (FTLD-TDP) inclusions are distinct proteinopathies that frequently cause similar frontotemporal dementia (FTD) clinical syndromes. FTD syndromes often display macroscopic signatures of neurodegeneration at the level of regions and networks, but it is unclear if subregional laminar pathology display patterns unique to proteinopathy or clinical syndrome. We hypothesized that FTLD-tau and FTLD-TDP accumulate pathology in relatively distinct cortical layers independent of clinical syndrome, with greater involvement of lower layers in FTLD-tau. The current study examined 170 patients with either FTLD-tau (*n* = 73) or FTLD-TDP (*n* = 97) spanning dementia and motor phenotypes in the FTD spectrum. We digitally measured the percent area occupied by tau and TDP-43 pathology in upper layers (I–III), lower layers (IV–VI), and juxtacortical white matter (WM) from isocortical regions in both hemispheres where available. Linear mixed-effects models compared ratios of upper to lower layer pathology between FTLD groups and investigated relationships with regions, WM pathology, and global cognitive impairment while adjusting for demographics. We found lower ratios of layer pathology in FTLD-tau and higher ratios of layer pathology in FTLD-TDP, reflecting lower layer-predominant tau pathology and upper layer-predominant TDP-43 pathology, respectively (*p* < 0.001). FTLD-tau displayed lower ratios of layer pathology related to greater WM tau pathology (*p* = 0.002) and to earlier involved/severe pathology regions (*p* = 0.007). In contrast, FTLD-TDP displayed higher ratios of layer pathology not related to either WM pathology or regional severity. Greater cognitive impairment was associated with higher ratios of layer pathology in FTLD-tau (*p* = 0.018), but was not related to ratios of layer pathology in FTLD-TDP. Lower layer-predominant tau pathology and upper layer-predominant TDP-43 pathology are proteinopathy-specific, regardless of clinical syndromes or regional networks that define these syndromes. Thus, patterns of laminar change may provide a useful anatomical framework for investigating how degeneration of select cells and corresponding laminar circuits influence large-scale networks and clinical symptomology in FTLD.

**Supplementary Information:**

The online version contains supplementary material available at 10.1007/s00401-021-02402-3.

## Introduction

The two most common types of proteinopathies in frontotemporal lobar degeneration (FTLD) are characterized by tau (FTLD-tau) and transactive response DNA-binding protein of 43 kDa (TDP-43) (FTLD-TDP) pathology [22, 57, 58]. FTLD proteinopathies are associated with a diverse spectrum of clinical presentations that include dementia phenotypes (i.e., behavioral variant FTD [bvFTD], primary progressive aphasia [PPA]) and motor phenotypes (i.e., amyotrophic lateral sclerosis [ALS], corticobasal syndrome [CBS], progressive supranuclear palsy syndrome [PSPS]) [42]. Accurate antemortem diagnosis of underlying neuropathology still remains a major challenge [59, 67], especially in bvFTD which has an equal likelihood of being caused by FTLD-tau or FTLD-TDP. MRI studies find that FTD syndromes caused by disparate pathologies often share patterns of cortical atrophy and aberrant functional connectivity in defined cognitive networks [76, 80, 81, 92, 104]. Since FTLD-type tau or TDP-43 pathology are not yet reliably detected in living patients, it remains unclear how dissimilar proteinopathies cause similar clinical phenotypes. However, detailed postmortem studies of human brains may help elucidate the microanatomy of overlapping clinical heterogeneity in FTLD and guide biomarker development.

Histopathologic studies can test predictions made by models of neural network degeneration that investigate how FTLD-tau and FTLD-TDP patients may present with similar clinical syndromes [1, 52, 77, 88, 101, 104]. One hypothesis to explain clinical similarity in FTLD is that tau and TDP-43 pathologic aggregates are anatomically concordant and disrupt the same networks. This implies shared anatomical vulnerability at a microscopic level (e.g., cortical neurons), mesoscopic level (e.g., neural circuits), or macroscopic level (e.g., cortical regions). Indeed, histopathologic studies of bvFTD have shown that select cell populations may contribute to salience network dysfunction [47, 74, 89] after accruing either tau [55, 90] or TDP-43 pathology [68]. An alternative hypothesis is that the same clinically relevant networks are disrupted by different types of neurons, circuits, or regions [21, 88, 101, 104]. In support of this latter hypothesis, recent investigations by our lab and others have found different regional patterns of peak tau and TDP-43 histopathologic burden within the language network in PPA [29, 27, 48, 49] and frontotemporal networks in bvFTD [43, 45]. FTLD proteinopathies may also display different laminar distributions of pathology in the same clinical syndrome and network, but comparative examinations of tau and TDP-43 pathology in cortical laminae are lacking.

Laminar cytoarchitecture influences whole-brain network connectivity [35] through diverse neural circuits (e.g., excitatory vs. inhibitory) that originate from select upper layers (I–III) and lower layers (IV–VI) [2, 13–15, 33, 32, 84]. Additionally, heterogeneous isocortical regions with less differentiation (fewer cortical layers) or more differentiation (more cortical layers) likely affect structural connectivity, functional activity, and potentially spread of FTLD pathology [13–15, 32, 26, 35, 36]. Therefore, cortical layers represent an anatomical paradigm for identifying new patterns of histopathologic accumulation within cells and neural circuits poorly understood in the FTLD spectrum. Primary tauopathies (i.e., Pick’s disease [PiD], corticobasal degeneration [CBD], and progressive supranuclear palsy [PSP]) produce widespread tau pathology in most cortical layers, but lower layers often accumulate more Pick cells in PiD, ballooned neurons in CBD, and globose tangles in motor cortex of PSP [4– 7, 34, 37, 38, 97, 98]. In contrast, most TDP-43 proteinopathies accumulate TDP-43 inclusions in upper layers with varied involvement of lower layers [8, 9, 22, 53, 56–58, 70, 71]. While these studies suggest that select inclusions accumulate according to cortical layers and provide criteria for subtype classifications, many were limited by their focus to relatively small groups of patients that did not encompass the full pathologic, genetic, and clinical heterogeneity associated with FTLD. As a result, the potential influence of demographics, clinical features, and regions on total pathologic burden across cortical layers in the FTLD spectrum remain poorly understood. Given clinical similarities between FTLD-tau and FTLD-TDP patients, comparative histopathologic studies of FTLD-tau and FTLD-TDP offer a unique clinical–anatomical–pathological framework for understanding mechanisms by which common networks degenerate due to separate proteinopathies.

In this study, we examined the full clinicopathologic spectrum of FTLD to test the hypothesis that severe lower layer pathology is a distinguishing feature of FTLD-tau compared to FTLD-TDP. We calculated ratios of cortical layer pathology to establish a common metric of laminar distributions of pathology, thereby allowing direct comparisons of FTLD groups known to differ in total pathologic burden [22]. Ratios of layer pathology were measured in heterogeneous regions from distinct pathologic and clinical subgroups of FTLD and compared to WM pathology, disease severity, and cognitive impairment to elucidate patterns of potential progression and clinical relevance. Our comparative investigation indicates that TDP-43 pathology is consistently upper layer-predominant among TDP-43 proteinopathies, while tau pathology is more often lower layer-predominant among tauopathies, independent of region or clinical presentation. Furthermore, we provide new evidence that tau and TDP-43 pathology form different laminar distributions in the same dementia phenotypes and that upper layer pathology may contribute to global cognitive and behavioral deficits. These signature laminar patterns in separate FTLD proteinopathies may reveal distinct mechanisms of neurodegeneration in future work.

## Materials and methods

### Patients

Patients were clinically followed at the Frontotemporal Degeneration Center, Alzheimer’s Disease Research Center, Comprehensive Amyotrophic Lateral Sclerosis Center, or Movement Disorder Center at the University of Pennsylvania (Penn). Clinical diagnoses were designated prospectively based on published consensus guidelines [10, 31, 40, 61, 82]. Patients evaluated prior to modern clinical criteria were assigned clinical phenotypes based on retrospective chart review and consensus by experienced clinicians and researchers (M.G., D.J.I., C.T.M., L.M., K.R., D.A.W.). Brain autopsies were performed at the Hospital of University of Pennsylvania and the Penn Center for Neurodegenerative Disease Research. As described previously [95], fresh tissue was sampled at autopsy and fixed overnight in 10% neutral buffered formalin or 70% ethanol with 150 mmol NaCl. Neuropathologic diagnoses were made by neuropathologists (E.B.L. and J.Q.T.) using established criteria [22, 57, 60, 66]. All procedures in this study were performed in accordance with the standards of the Penn Institutional Review Board and the Declaration of Helsinki. Patient data were retrieved from the Penn Integrated Neurodegenerative Disease Database [103].

The current study included patients with a primary neuropathological diagnosis of either FTLD-tau (*n* = 73) or FTLD-TDP (*n* = 97). The FTLD-tau group included PiD (*n* = 14), CBD (*n* = 14), PSP (*n* = 36), and unclassifiable tauopathies (TauU, *n* = 9), the latter included individuals with either unique tau accumulations (*n* = 3) or *MAPT* mutations (*n* = 6). The FTLD-TDP group included FTLD-TDP types A (*n* = 25), B (*n* = 17), C (*n* = 17), and E (*n* = 5), in addition to amyotrophic lateral sclerosis (ALS, *n* = 34) comprising patients with motor-predominant ALS (*n* = 28) or ALS with FTD (*n* = 6). Patients were prospectively genotyped for pathogenic mutations on *MAPT, C9orf72, GRN,* and other FTD-associated genes based on a structured pedigree analysis described previously [102]. Carriers of *C9orf72* (*n* = 21), *GRN* (*n* = 14), *TBK1* (*n* = 2), and *GBE1* (*n* = 1) mutations met standard criteria for FTLD-TDP subtype classification [57]. Some FTLD patients had secondary pathologic diagnoses that largely involved brain regions outside the cortical areas examined in the current study. Patient demographics and pathologic characteristics are summarized in Table 1.

**Table 1:** Patient demographics and pathologic characteristics

FTLD group	(*N*)	Sex	(*N*)	Education (years)	Age at death (years)	Disease Duration (years)	Post-mortem Interval (hours)	Mutation status	(*N*)	Primary Neuro-pathologic Diagnoses	(*N*)	Co-morbid pathologies				Clinical diagnoses	(*N*)
												Secondary Neuro-pathologic Diagnoses	(*N*)	AD Neuro-pathologic Change	***(****N****)***		
tau	73	Female	33	15.3 (2.6)	71 (11.1)	7.9 (3.8)	11.7 (7.2)	MAPT	6	CBD	14	ARTAG	28	Not	39	bvFTD	28
		Male	40	[10–20]	[31–94]	[3–20]	[1.5–41]			PiD	14	HS	1	Low	26	CBS	5
					*	*				PSP	36	LBD-A	1	Int	4	PPA	16
										TauU	9	LBD-B	8	High	2	PSPS	24
														N/A	2		
TDP	97	Female	41	15.6 (3.0)	66 (9.7)	6 (3.6)	11.8 (6.8)	C9orf72	21	ALS	34	ARTAG	16	Not	25	ALS	28
		Male	56	[10–22]	[41–96]	[1–19]	[0–30]	GBE1	1	TDP-A	25	CVD	2	Low	41	bvFTD	50
								GRN	14	TDP-B	17	CTE	1	Int	8	CBS	2
								TBK1	2	TDP-C	16	HS	7	High	2	PPA	17
										TDP-E	5	LBD-A	5	N/A	0		
												LBD-B	7				
												LBD-L	3				
												PART	21				
												TauU	3				

### Clinical data

To obtain a harmonized global measure of cognitive impairment available in the majority of our cohort spanning decades of brain banking, we focused on Mini-Mental State Examination (MMSE) scores collected from Penn INDD [24]. The relationship between laminar pathology and MMSE performance was tested using cross-sectional MMSE scores closest to death (within 4 years), which were available from a majority of FTLD-tau (*n* = 40) and FTLD-TDP (*n* = 56) patients. Total MMSE score ranges from unimpaired (30) to severely impaired (0) and used as a continuous measure in analyses below. To explore clinicopathologic relationships further, we compared laminar distributions of pathology to a validated measure of the severity of behavioral disturbances based on the Neuropsychiatric Inventory Questionnaire (NPI-Q) [23, 46]. Cross-sectional NPI-Q data closest to death (within 4 years) were available in a subset of our cohort (13 FTLD-tau, 15 FTLD-TDP).

### Isocortical regions of interest

**Fig. 1:**
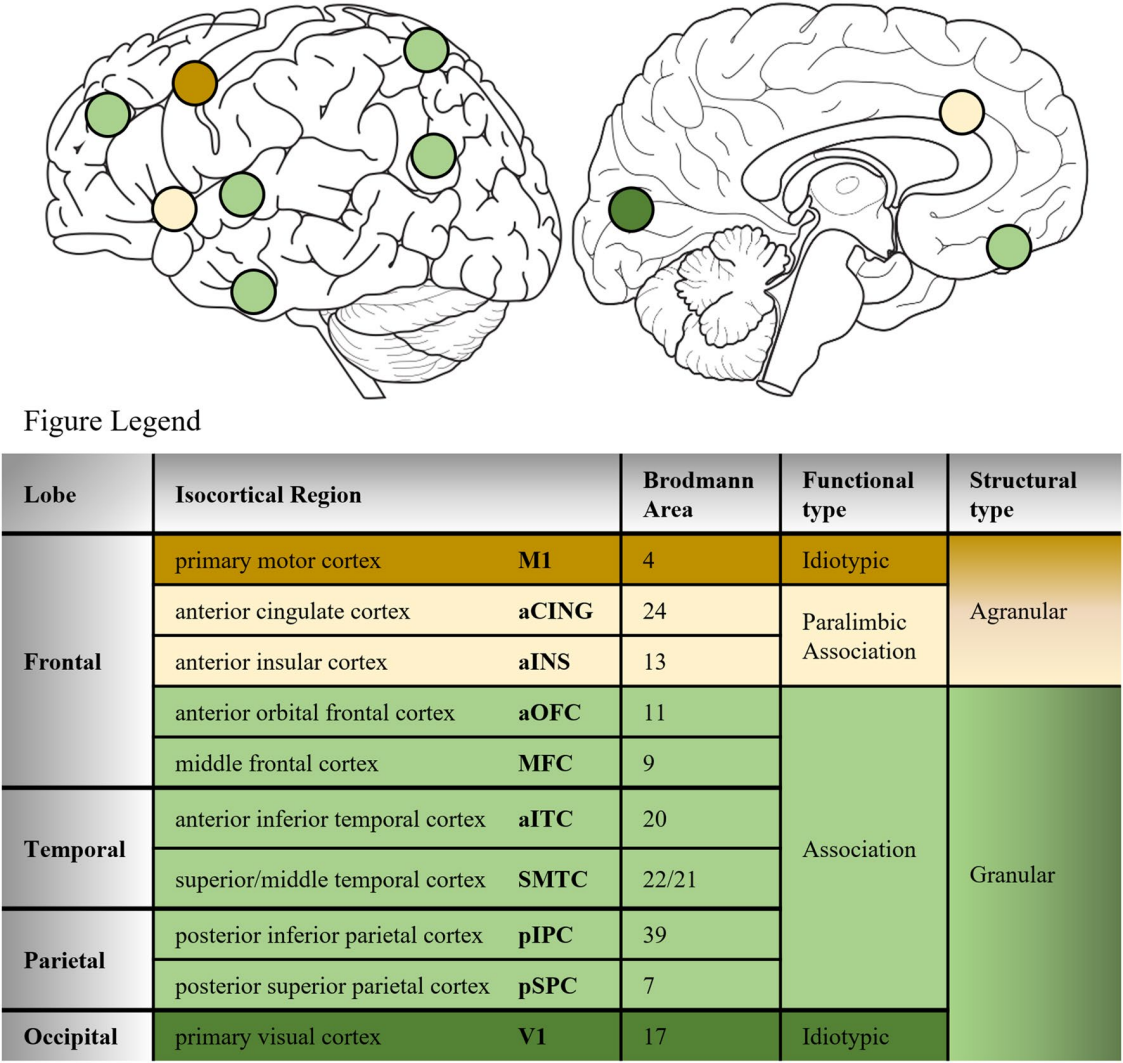
Isocortical regions of interest. Laminar distributions of pathology were quantified in ten isocortical regions associated with different lobes, functions, and structural cytoarchitecture. Agranular cortices included paralimbic association regions and idiotypic primary motor cortex (dark brown). Granular cortices included association regions (light green) and more distinct granular cortex (koniocortex) included idiotypic primary visual cortex (dark green)

The current investigation quantified laminar and WM pathology in ten isocortical regions (Fig. 1). The frequency of all regions examined in FTLD groups and subgroups are summarized in Supplementary Table 1 (online resource) including bilateral regions available from select frontal, temporal, and parietal regions in 33 brain autopsies. Functionally, we examined regions that belong to either idiotypic (i.e., M1 and V1) or association cortices, the latter referring to regions inside (i.e., aCING and aINS) or outside (i.e., aOFC, MFC, aITC, SMTC, pIPC) the paralimbic zone that subserve different cognitive and behavioral functions [62, 63]. Structurally, these regions have laminar organizations categorized as either agranular cortex (absent or inconspicuous layer IV) or granular/eulaminate cortex (six distinct layers or more) as summarized in Fig. 1 [14, 26].

Previous investigations of select tauopathies (i.e., PiD, PSP) and TDP-43 proteinopathies (i.e., ALS, FTLD-TDP types A-C) have identified regional patterns of tau and TDP-43 pathology that may reflect stages of pathologic propagation between connected regions [18–20, 43, 50]. Thus, to examine patterns of potential pathologic progression across layers, we compared ratios of layer pathology between regions classified by their stage of involvement (earlier vs. later involved designations determined previously) [18–20, 43, 50] or severity of total GM pathology (i.e., severe vs. mild designations determined by a region’s median burden falling above or below the median burden of the total pathologic subgroup) (Supplementary Tables 2–3, online resource).

Given that multiple regions comprise distinguishable neural networks differentially involved in clinical syndromes such as bvFTD and PPA, we examined laminar patterns in regions part of the paralimbic salience network (bilateral aCING and aINS), the executive-control network (bilateral MFC and pSPC), and the language network (left MFC, aITC, SMTC, and pIPC) [76, 85, 89, 91, 92].

### Immunohistochemistry

Immunohistochemistry procedures for regions sampled above were completed in the Penn Digital Neuropathology Lab and described in detail previously [29, 72]. In brief, paraffin-embedded 6 µm-thick sections were immunostained for either phosphorylated tau (mouse monoclonal antibody, pTau (S202/T205), clone AT8, 1:1000, Invitrogen) in the FTLD-tau group [17, 22] or phosphorylated TDP-43 (rat monoclonal antibody, pTDP-43 (S409/410), clone 1D3, 1:1000, MilliporeSigma) in the FTLD-TDP group [69]. All immunostained tissue was counterstained with fresh hematoxylin for the visualization of cell organization (i.e., cell density, morphology, and size), providing a reliable means to identify boundaries between cortical layers and WM in all regions (see details below).

### Laminar regions of interest and digital image approach

Immunostained tissue sections were imaged on a digital slide scanner (Aperio AT2, Leica Biosystem, Wetzlar, Germany) in the Penn Digital Neuropathology Lab at 20 × magnification. Images were digitally analyzed using QuPath software (version 0.2.0). Pathologic burden was measured as the percent area occupied (%AO) by tau- or TDP-43-immunoreactive pixels in three subregions per section. Positive pixel classifiers were empirically derived for each batch of immunostained sections in FTLD-tau and FTLD-TDP as previously validated [28].

An experienced neuroanatomist (D.T.O.) delineated the three subregions per section (i.e., upper layers I–III, lower layers IV–VI, and juxtacortical WM) based on regional cytoarchitecture (Supplementary Fig. 1, online resource). An unbiased view of the cytoarchitecture blinded to any immunoreactive inclusions that may be present in the field of view was created in QuPath by closing all color channels except the blue hematoxylin channel. Next, using a belt-transect method of sampling in each section [3, 44], three neighboring subregions orthogonal to the pial surface and ~ 1 mm wide were manually annotated in the longest stretch of relatively flat isocortex (typically sulcal walls). This systematic sampling of gray matter avoided poorly represented cortical layers due to cortical folds or oblique cuts through gyri [84, 99].

Upper and lower layer subregions were differentiated according to laminar patterns of cellular organization (i.e., cellular size and density) specific to each isocortical region. For agranular regions with little to no layer IV (M1, aCING, and aINS), upper and lower layers were distinguishable by the relatively smaller pyramidal neurons of layer III abutting the larger pyramidal neurons of layer V. For granular/eulaminate regions, the border between upper and lower layers was reliably identified by the relatively lower density of larger, mostly pyramidal neurons of layer III adjacent to the higher density of smaller, mostly non-pyramidal neurons of layer IV. In all regions, the border between layer VI and WM was consistently determined by the distinctly larger polymorphic neurons of layer VI adjacent to the higher density of smaller oligodendrocytes comprising WM. We excluded tissue with artifacts or tears that prevented the examination of cytoarchitecture and segmentation of all cortical laminae. In total, 1083 tissue sections met inclusion criteria for laminar analyses in the current study (Supplementary Table 1, online resource).

### Statistical analyses

Total gray matter (GM) pathologic burden was measured as the average %AO by pathology in combined cortical layers. We calculated ratios of upper to lower layer pathology and ratios of GM-to-WM pathology per section to preserve intrinsic pathologic distributions per patient and for direct comparisons between FTLD-tau and FTLD-TDP groups. Ratios of GM-to-WM pathology included the total GM pathologic burden in all cortical layers combined. GM pathology, WM pathology, and ratios of pathology underwent natural log (ln) transformations to normalize data used in all analyses. The normalized layer ratio data produced positive values (upper layer-predominant pathology), negative values (lower layer-predominant pathology), or ratios approximately zero (bilaminar pathology). The normalized GM-to-WM ratio data produced positive or negative values that represented GM-predominant or WM-predominant pathology, respectively.

Demographics were compared using Chi-square analyses for categorical data and Wilcoxon Mann–Whitney U tests for non-parametric continuous data. Laminar distributions of pathology in FTLD-tau and FTLD-TDP were analyzed using linear mixed-effects (LME) models that included ratio of layer pathology as the dependent variable and random intercepts for individual patients to account for repeated measures and missing data. All LME models included hemisphere, region, age at death, and disease duration as fixed-effects and covariates to adjust for their potential contributions to the dependent variable (i.e., total GM pathology, ratio of layer pathology, or ratio of GM-to-WM pathology). Select analyses included additional fixed-effects and covariates as indicated below. Missing data due to tissue unavailability or poor integrity were accounted for in all models and results. All statistical tests were 2-sided and *p* < 0.05 was considered significant. All analyses were performed using SPSS (version 27.0; SPSS, Chicago, IL).

### Comparisons of laminar pathology between FTLD groups and subgroups

First, a LME model compared ratios of layer pathology by FTLD group (i.e., FTLD-tau and FTLD-TDP). To ensure concomitant pathologies did not account for differences in laminar pathology, analyses were repeated excluding patients with co-occurring pathologies. To confirm consistency of ratios of layer pathology within each FTLD group, pathologic subgroup (i.e., PiD, CBD, PSP, TauU in FTLD-tau; TDP A-E, ALS in FTLD-TDP) was first included as a fixed-effect in separate LME models for each FTLD group. Second, genetic subgroup (i.e., sporadic vs. familial) was included as a fixed-effect in separate LME models for each FTLD group. Third, clinical subgroup (i.e., bvFTD, PPA, CBS, PSPS, ALS) was included as a fixed-effect in separate LME models for each FTLD group, with Bonferroni corrected pair-wise post hoc comparisons of main effects using estimated marginal means.

### Comparisons of laminar pathology between FTLD-tau and FTLD-TDP in select regions

To confirm variations in regional microstructure and function did not account for differences in laminar pathology, ratios of layer pathology between total FTLD-tau and FTLD-TDP were compared using three LME models in regions grouped by idiotypic M1, paralimbic association, or association cortex. Idiotypic V1 was a unilaterally sampled control region, thus linear regression compared the ratio of layer pathology in V1 to total FTLD-tau and FTLD-TDP, controlling for age at death and disease duration.

### Assessments of hypothesized progression of laminar pathology in FTLD-tau and FTLD-TDP

Hypothesized disease progression across regions was tested using separate LME models for each FTLD group that included severe/mild pathology regions as a fixed-effect and total GM pathology as the dependent variable. These models were repeated using earlier/later involved regions as a fixed-effect and total GM pathology as the dependent variable. Next, separate LME models for each total FTLD group compared ratios of layer pathology between severe/mild pathology regions. Similarly, separate LME models for each total FTLD group compared ratios of GM-to-WM pathology between severe/mild pathology regions. These analyses were repeated in earlier/later involved regions for ratios of layer pathology and ratios of GM-to-WM pathology in each FTLD group. Finally, separate LME models for each total FTLD group tested ratios of layer pathology as a function of juxtacortical WM pathology.

### Relationships between laminar pathology and clinical assessments of cognition and behavior in FTLD-tau and FTLD-TDP

First, separate LME models for each total FTLD group tested total GM pathology as a function of the continuous variable final MMSE score. Next, separate LME models for each total FTLD group tested ratios of layer pathology as a function of final MMSE score or final NPI-Q score. Finally, given that the MMSE may better detect cognitive impairment in bvFTD compared to other non-amnestic phenotypes [73], separate LME models for bvFTD-tau and bvFTD-TDP tested ratios of layer pathology as a function of final MMSE score (fixed-effect). All LME models that examined clinicopathologic relationships included the following fixed-effects/covariates: hemisphere, region, sex, education, age at death, and time interval between final clinical assessment and death.

### Comparisons of laminar pathology between FTLD-tau and FTLD-TDP in bvFTD

We repeated analyses of ratios of layer pathology in bvFTD and PPA syndromes caused by changes to regions part of distinct cognitive networks. First, separate LME models for bvFTD and PPA compared ratios of layer pathology between FTLD-tau and FTLD-TDP. Second, we accounted for clinically relevant regions in each clinical phenotype in the following models: one LME model compared ratios of layer pathology between FTLD-tau and FTLD-TDP in bilateral salience/executive-related regions relevant to bvFTD, and another LME model compared ratios of layer pathology between FTLD-tau and FTLD-TDP in left language-related regions relevant to PPA.

## Results

### Patient demographics

FTLD groups were well-matched across multiple demographics and pathologic features (Table 1). On average, the total FTLD-tau group was older (*Z* = − 3.14, *p* = 0.002) and had a longer disease duration (*Z* = − 2.91, *p* = 0.004) than the total FTLD-TDP group.

### Distinct laminar distributions of pathology in FTLD-tau and FTLD-TDP

**Fig. 2:**
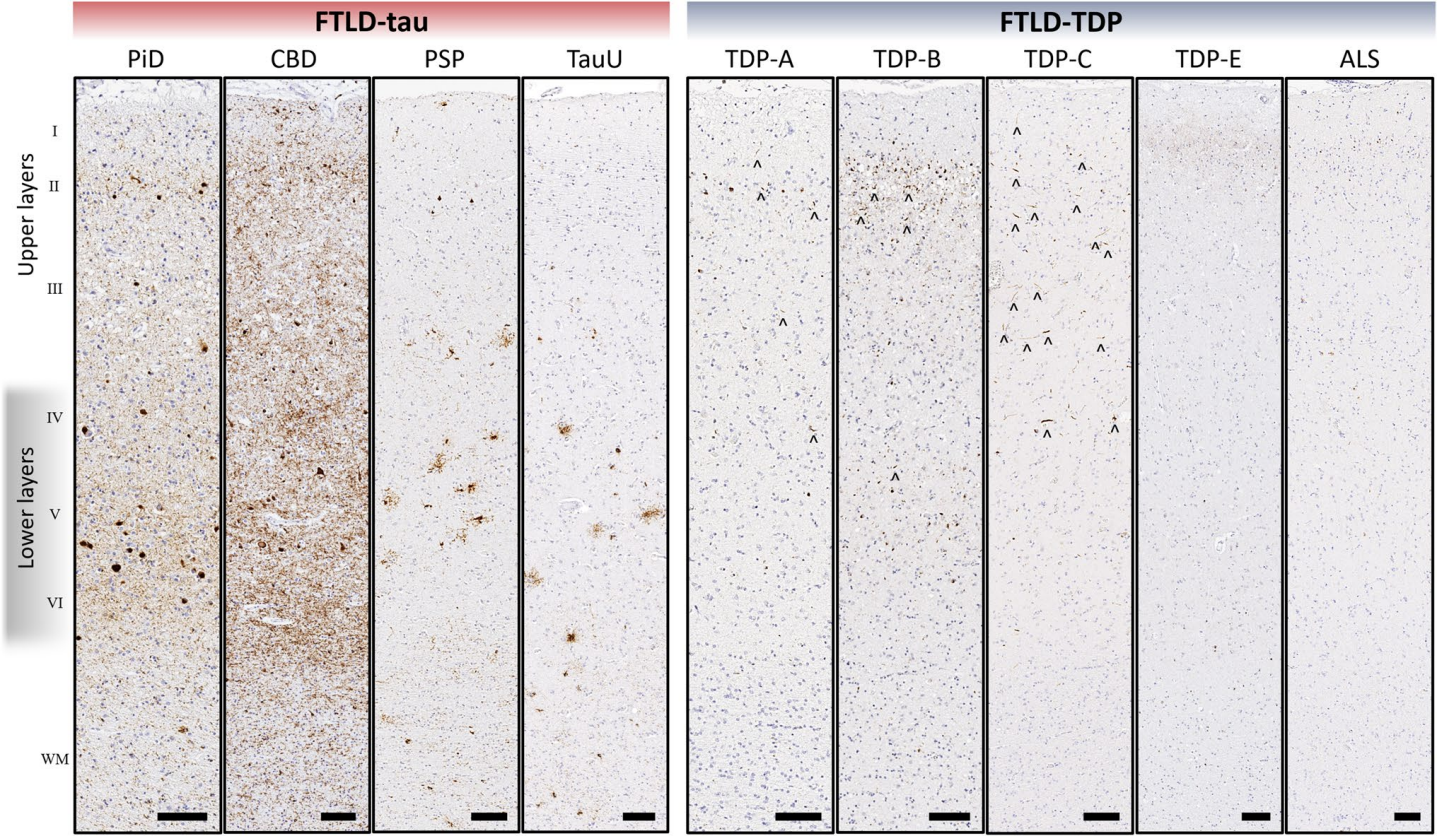
Representative distributions of tau and TDP-43 pathology in cortical layers and juxtacortical white matter in pathologic subgroups of FTLD-tau and FTLD-TDP. FTLD-tau typically displayed a lower layer-predominant profile of AT8-immunoreactive tau pathology, while FTLD-TDP displayed an upper layer-predominant profile of 1D3-immunoreactive TDP-43 pathology. Neuronal and glial inclusions were interspersed among cortical layers and consistent with each pathologic subtype. In FTLD-tau, neuronal inclusions (i.e., pre-tangles, tangles, globose tangles, ballooned neurons) and glial inclusions (i.e., tufted astrocytes, astrocytic plaques, ramified astrocytes, coiled bodies) were found in all cortical layers in most regions examined, with high densities of neuritic threads common to lower layers in many regions. WM tau accumulation was often similar to total GM tau burden, and generally resembled neuritic threads or coiled bodies. In FTLD-TDP, TDP-43 pathology was observed more often in upper layers and was comprised of mostly small neuronal cytoplasmic inclusions and dystrophic neurites (open arrows point to examples of small neurites) depending on the pathologic subtype of TDP. TDP-43 pathology in WM typically resembled curvilinear oligodendrocytic inclusions that were consistently less frequent than TDP-43 pathology in GM. All images acquired from the MFC region. Roman numerals indicate cortical layers. Scale bars = 100 µm

**Fig. 3:**
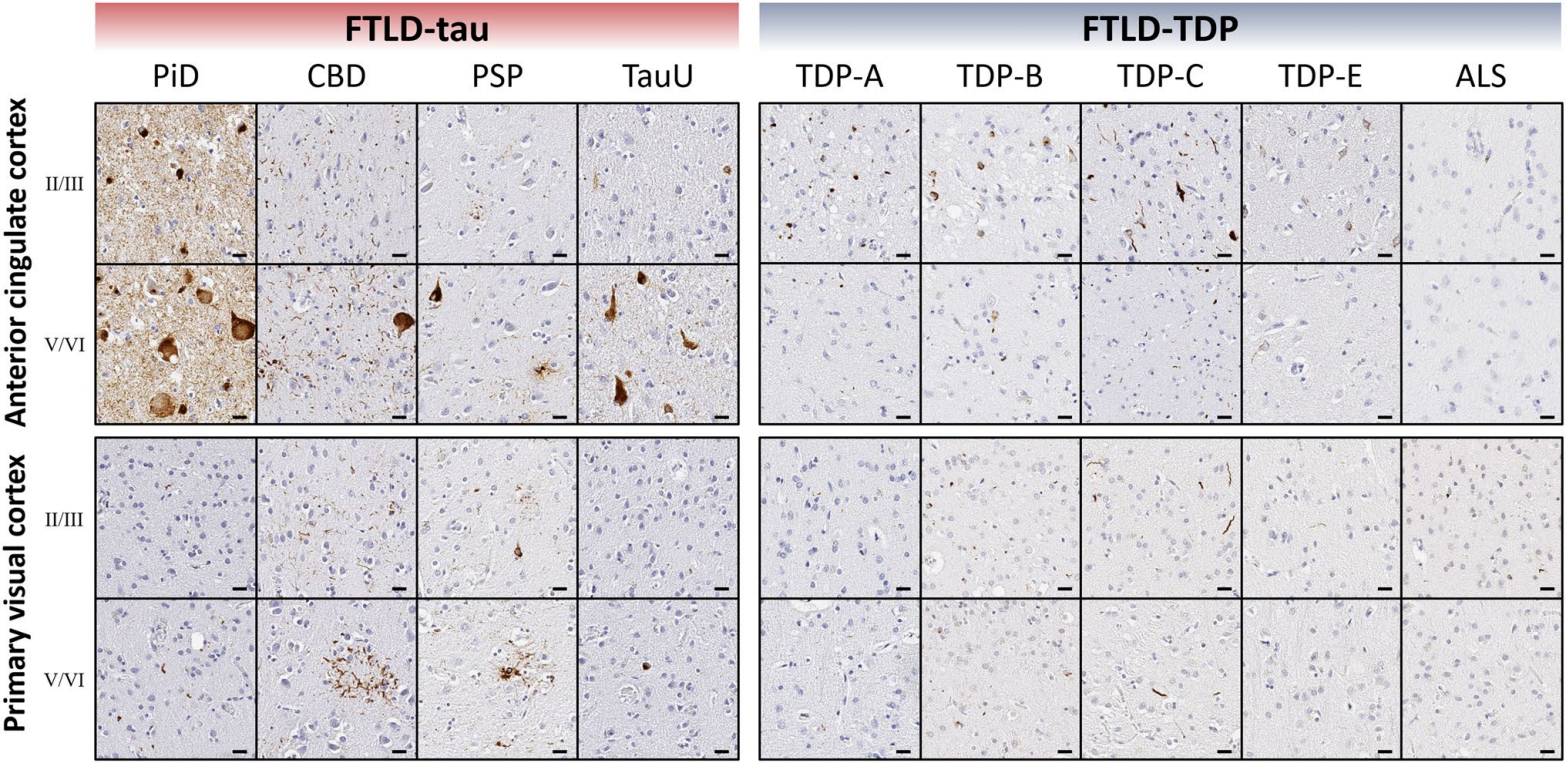
Representative photomicrographs of tau and TDP-43 pathology in supragranular and infragranular layers of select regions. Distinct laminar distributions of tau and TDP-43 pathology were largely consistent across regions examined, despite regional differences in cytoarchitecture, function, or total pathologic burden. Roman numerals indicate cortical layers. Scale bars = 20 µm

**Fig. 4:**
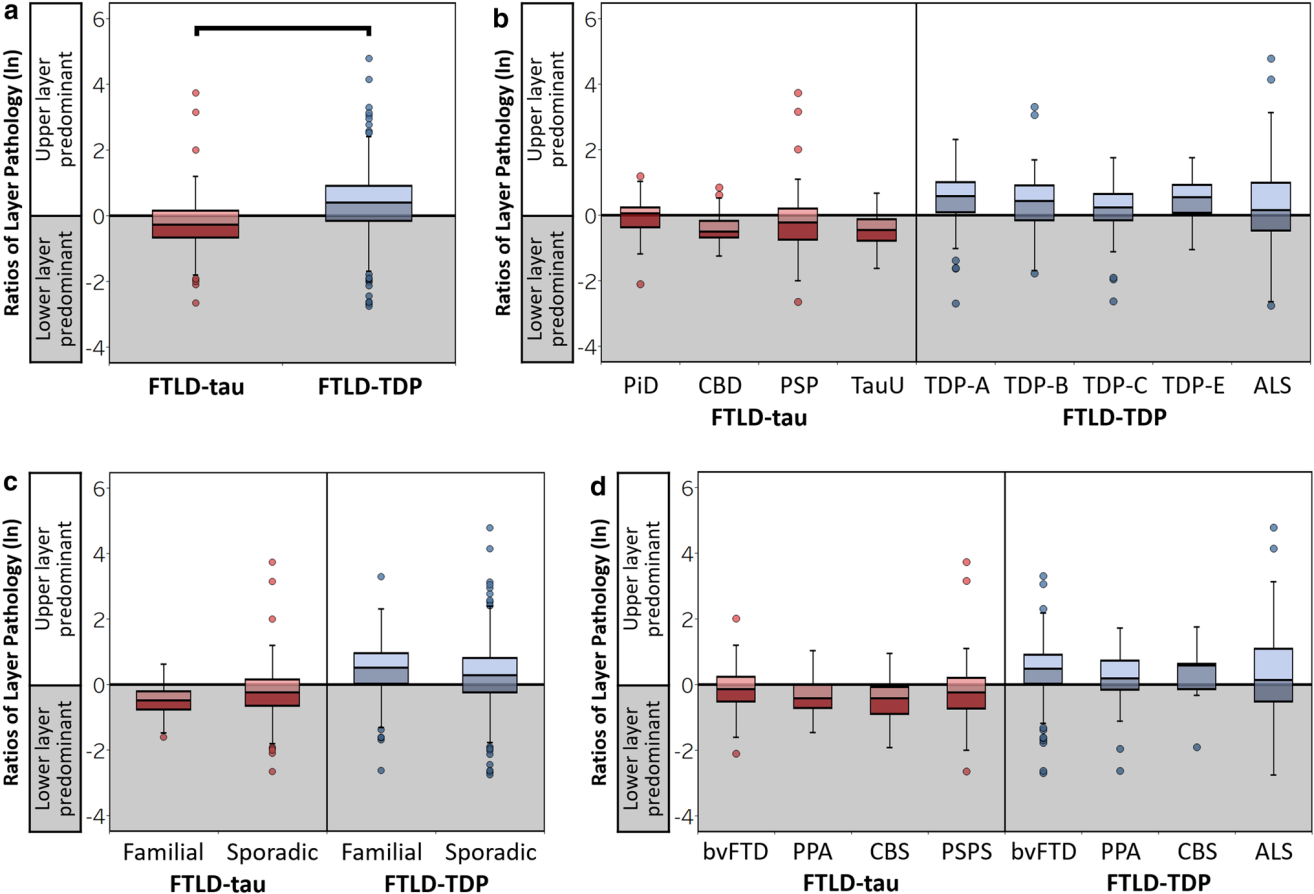
Laminar distributions of tau and TDP-43 pathology are distinct and consistent between pathologic, genetic, and clinical subgroups. Ratios of layer pathology by total FTLD groups (**a**). FTLD-tau was associated with a significantly lower ratio of layer pathology (lower layer-predominant tau pathology) compared to the higher ratio of larger pathology (upper layer-predominant TDP-43 pathology) in FTLD-TDP (*β* = -0.61, SE = 0.07, *p* < 0.001). Within-group comparisons of ratios of layer pathology by pathologic (**b**), genetic (**c**), and clinical subgroups (**d**) demonstrate that distinct ratios of layer pathology are largely similar between subgroups of FTLD-tau and between subgroups of FTLD-TDP. Boxplots represent ratios of layer pathology per region of each patient

TDP-43 pathology was consistently observed in upper layers with relatively less involvement of deeper cortex across FTLD-TDP subgroups. In contrast, tau pathology was more frequent and widespread across cortical layers, with prominent involvement of lower layers and WM across FTLD-tau subgroups (Figs. 2, 3). To quantitatively determine if tau and TDP-43 pathology accumulated unique patterns across cortical laminae, we first compared ratios of layer pathology between total FTLD-tau and FTLD-TDP groups. We found that FTLD-tau was associated with a significantly lower ratio of layer pathology compared to the higher ratio of layer pathology in FTLD-TDP (*β* = − 0.62, SE = 0.07, *p* < 0.001), indicating relatively greater tau burden in lower layers compared to relatively greater TDP-43 burden in upper layers (Fig. 4a). In this model, all other effects were non-significant (*p* > 0.7) except region (*p* = 0.003).

We replicated these findings in a subset of patients with no missing data (i.e., complete data from the same frontal or temporal regions), suggesting that variations in region availability (missing data) between patients did not influence the protienopathy-specific laminar distributions of pathology we measured (Supplementary Fig. 3, online resource). Importantly, we found similar results when we excluded the ALS subgroup (*n* = 34) from the total FTLD-TDP group (*n* = 97), leaving only patients with TDP types A–E (*n* = 63). Specifically, we found that FTLD-tau still displays relatively greater lower layer pathology in comparison to the relatively greater upper layer pathology in clinically similar TDP types A–E (*β* = − 0.65, SE = 0.07, *p* < 0.001).

Next, we tested for consistency between pathologic, genetic, and clinical subgroups within FTLD-tau and FTLD-TDP (Fig. 4b–d, Supplementary Table 4, online resource). Pathologic subgroups of FTLD-tau displayed similarly low ratios of layer pathology (*p* = 0.132) and pathologic subgroups of FTLD-TDP displayed similarly high ratios of layer pathology (*p* = 0.265) (Fig. 4b). Ratios of layer pathology did not differ between sporadic and familial subgroups of FTLD-tau (*p* = 0.637) or FTLD-TDP (*p* = 0.267) (Fig. 4c). Lastly, we found that ratios of layer pathology were largely consistent between clinical subgroups of FTLD-TDP (*p* = 0.540) and between clinical subgroups FTLD-tau (Fig. 4d, Supplementary Table 4, online resource).

To confirm that laminar patterns were not influenced by concomitant pathology (Table 1), we excluded patients with any pathology secondary to FTLD-tau and FTLD-TDP, including low-to-high AD neuropathologic change (ADNC). Similar to total group results, we found lower ratios of layer pathology in relatively “pure” FTLD-tau (*n* = 22) compared to “pure” FTLD-TDP (*n* = 22; *β* = − 0.60, SE = 0.08, *p* < 0.001) while all other effects were non-significant (*p* > 0.2).

### Distinct laminar distributions of pathology in FTLD-tau and FTLD-TDP are consistent across regions with varied cytoarchitecture and function

**Fig. 5:**
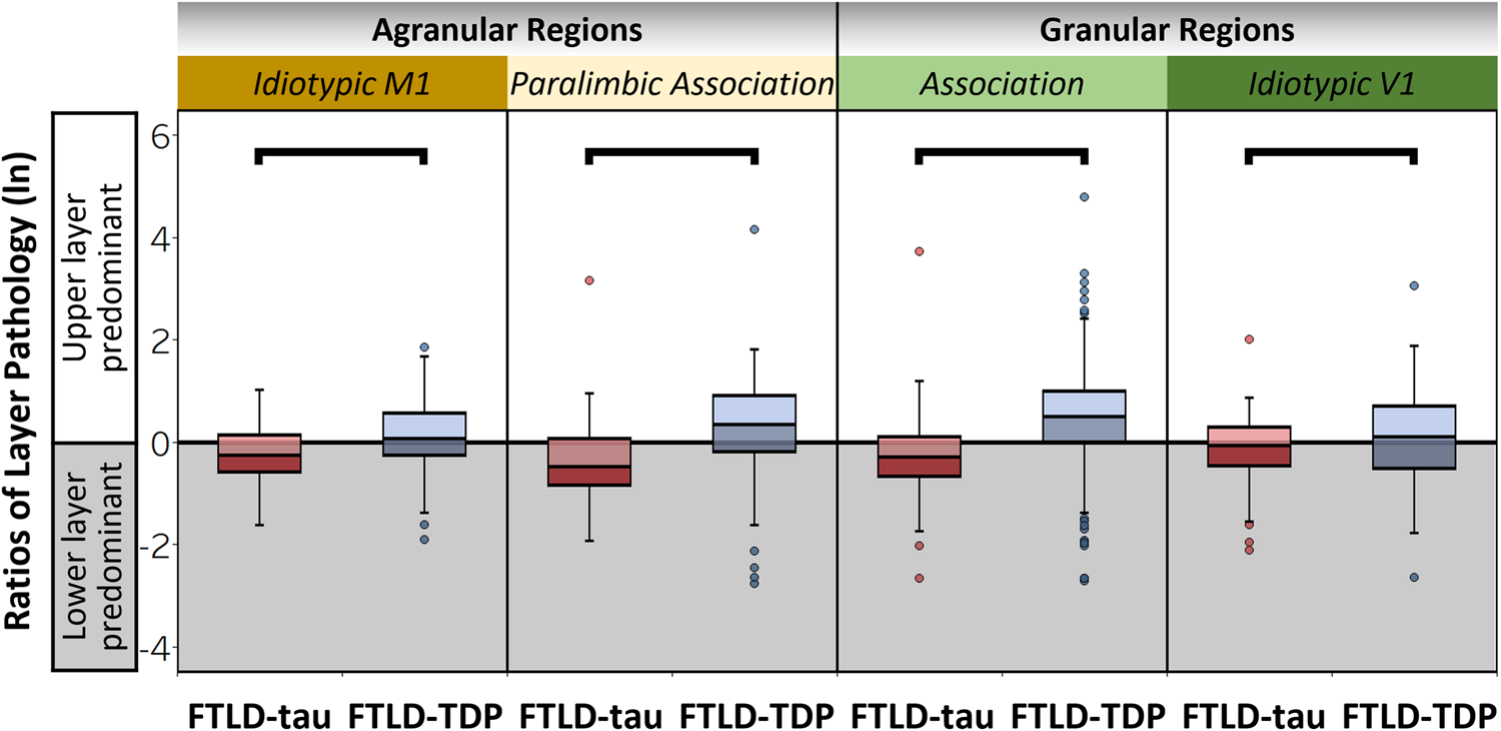
Distinct laminar distributions of tau and TDP-43 pathology are consistent across regions with varied cytoarchitecture and function. Ratios of layer pathology in regions with varied microstructure and function. Total FTLD-tau and total FTLD-TDP groups displayed significant differences in ratios of layer pathology in each type of agranular and granular region examined, including idiotypic M1 (*β* = -0.34, SE = 0.12, *p* = 0.004), paralimbic association regions (*β* = -0.81, SE = 0.19, *p* < 0.001), association regions (*β* = -0.71, SE = 0.08, *p* < 0.001), and idiotypic V1 (*β* = -0.29, SE = 0.14, *p* = 0.037). Boxplots represent ratios of layer pathology per region of each patient

We performed a series of regional analyses to determine if ratios of layer pathology differed by regions with different microstructure or function. In total FTLD groups, we analyzed regional subgroups based on microstructural and functional affiliations and consistently found distinct ratios of layer pathology between FTLD-tau and FTLD-TDP in idiotypic M1 (*β* = − 0.34, SE = 0.12, *p* = 0.004), paralimbic association (*β* = − 0.81, SE = 0.19, *p* < 0.001), association (*β* = − 0.72, SE = 0.08, *p* < 0.001), and idiotypic V1 (*β* = 0.30, SE = 0.14, *p* = 0.037) (Fig. 5). In these models, we observed a significant effect of region in paralimbic association regions (*p* = 0.047), while all other effects were non-significant (*p* > 0.05). These regional comparisons provide converging evidence that laminar distributions of pathology appear relatively unique to each proteinopathy across heterogeneous regions sampled.

### Laminar patterns of pathology in relation to regional disease severity and juxtacortical white matter in FTLD-tau and FTLD-TDP

**Fig. 6:**
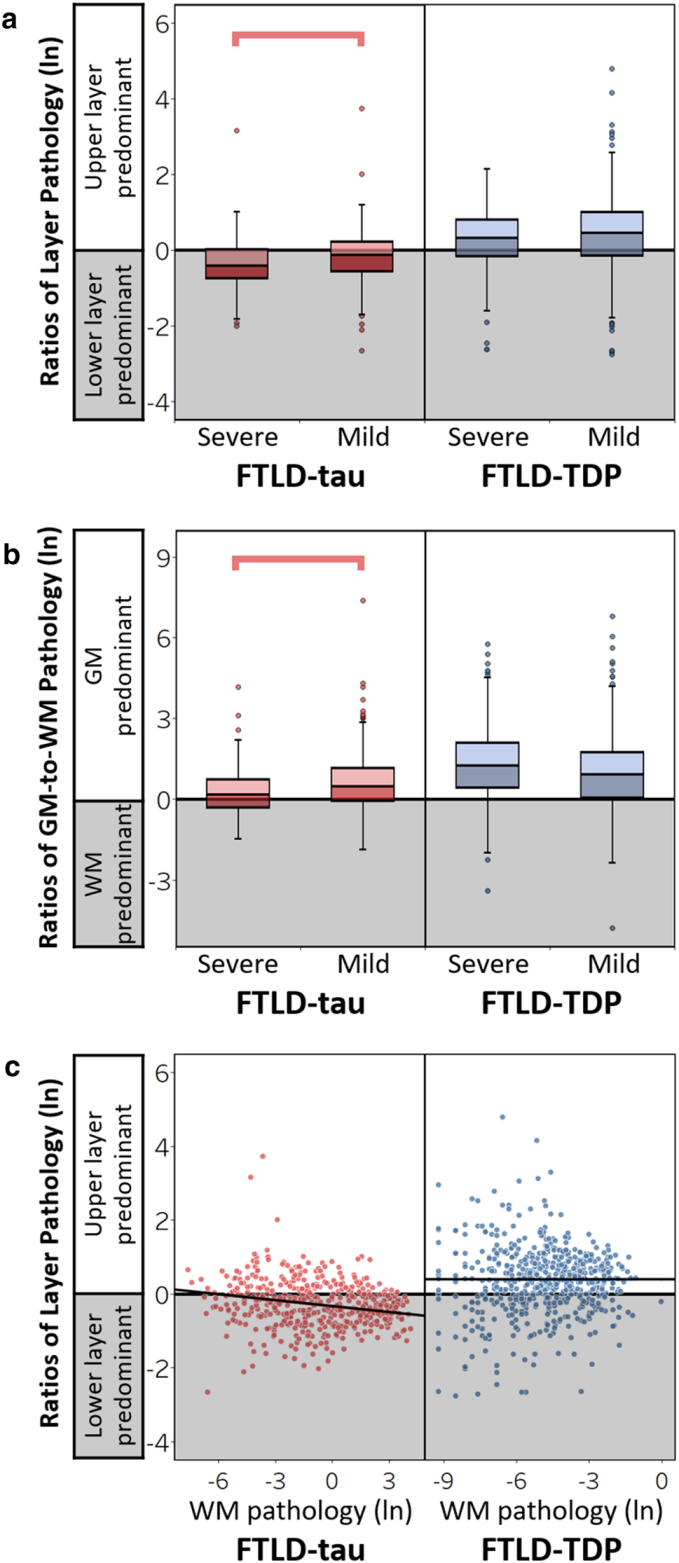
Laminar distributions of pathology in relation to regional disease severity and juxtacortical white matter in FTLD-tau and FTLD-TDP. **(a)** Ratios of layer pathology by regions with severe or mild pathologic burden in total FTLD-tau and FTLD-TDP. In FTLD-tau, severe pathology regions displayed lower ratios of tau pathology compared to mild pathology regions (*β* = -0.20, SE = 0.07, *p* = 0.006). In FTLD-TDP, however, higher ratios of layer pathology were similar between mild and severe pathology regions (*β* = -0.10, SE = 0.10, *p* = 0.309)**. (b)** Ratios of GM-to-WM pathology by regions with severe or mild pathologic burden in total FTLD-tau and FTLD-TDP. In FTLD-tau, severe pathology regions displayed lower ratios of GM-to-WM pathology compared to mild pathology regions (*β* = -0.34, SE = 0.10, *p* = 0.001). In FTLD-TDP, however, similar higher ratios of GM-to-WM pathology were displayed in severe and mild pathology regions (*β* = 0.17, SE = 0.14, *p* = 0.228). **(c)** Greater WM tau pathology was associated with lower ratios of layer pathology in FTLD-tau (*β* = -0.05, SE = 0.02, *p* = 0.001), whereas there was no relationship between WM TDP-43 pathology and the ratio of layer pathology in FTLD-TDP (*β* = -0.003, SE = 0.02, *p* = 0.90). Lines represent the predicted ratios of layer pathology as a function of WM pathology controlling for covariates in each model (i.e., hemisphere, region, age at death, and disease duration). Boxplots and scatterplots represent ratios of layer pathology per region of each patient

Because regions of different disease severity/stage of involvement may reflect patterns of pathologic spread [18–20, 43, 50], we tested the hypothesis that ratios of layer pathology may differ between these regions in FTLD-tau and FTLD-TDP. As expected, regions categorized as earlier involved or severe pathology had significantly greater total GM pathology compared to later involved or mild pathology regions, confirming that these approaches to categorizing regions may reflect disease progression (Supplementary Table 2–3, Supplementary Fig. 2a-b, online resources). Next, we compared ratios of layers pathology between regions categorized as severe or mild pathologic burden and found lower ratios of layer pathology in severe pathology regions compared to mild pathology regions (*β* = − 0.20, SE = 0.07, *p* = 0.007) in FTLD-tau (Fig. 6a) while all other effects were non-significant (*p* > 0.07). In contrast, relatively high ratios of layer pathology were not different between severe pathology and mild pathology regions (*β* = − 0.09, SE = 0.10, *p* = 0.362) in FTLD-TDP (Fig. 6a), indicating that TDP-43 pathology is largely restricted to upper layers throughout disease progression.

Similar to above, we tested the hypothesis that total GM pathology and laminar distributions of pathology may be related to juxtacortical WM pathology due to potential spread of pathology between the connected regions. In analyses of FTLD-tau, we found lower GM-to-WM ratios of tau pathology (greater WM-predominance) in severe pathology regions compared to mild pathology regions (*β* = -0.34, SE = 0.10, *p* = 0.001) while all other effects were non-significant (*p* > 0.09) except region (*p* = 0.021) (Fig. 6b). Additionally, greater WM tau pathology was associated with lower ratios of layer tau pathology (*β* = − 0.05, SE = 0.02, *p* = 0.002), while all other effects were non-significant (*p* > 0.3) except region (*p* = 0.048) (Fig. 6c). Analyses of FTLD-TDP produced distinct results from FTLD-tau: high ratios of GM-to-WM pathology (greater GM predominance) were not different between severe and mild pathology regions (*β* = 0.18, SE = 0.14, *p* = 0.199) (Fig. 6b), and WM TDP-43 pathology did not relate to ratios of layer TDP-43 pathology (*β* = − 0.00006, SE = 0.02, *p* = 0.998) in FTLD-TDP (Fig. 6c). Subanalyses controlling for pathologic subgroup produced similar findings. In FTLD-tau, pathologic subgroup was not a significant predictor (*p* = 0.081) and it did not influence the significant relationship between WM pathology and the ratio of layer tau pathology (*β* = − 0.06, SE = 0.02, *p* < 0.001). Similarly, in FTLD-TDP, pathologic subgroup was not a significant predictor (*p* = 0.210) and it did not influence the lack of relationship between WM pathology and the ratio of layer TDP-43 pathology (*β* = − 0.02, SE = 0.03, *p* = 0.370). Alternative categorization of regions into earlier vs. later stages of involvement produced similar results (Supplementary Fig. 3, online resource). These analyses provide converging evidence that TDP-43 pathology is consistently upper layer-predominant, whereas tau pathology is more pronounced in the lower layers and WM of severe pathology regions potentially earlier involved in disease progression.

### Clinicopathologic relationships in FTLD-tau and FTLD-TDP

**Fig. 7:**
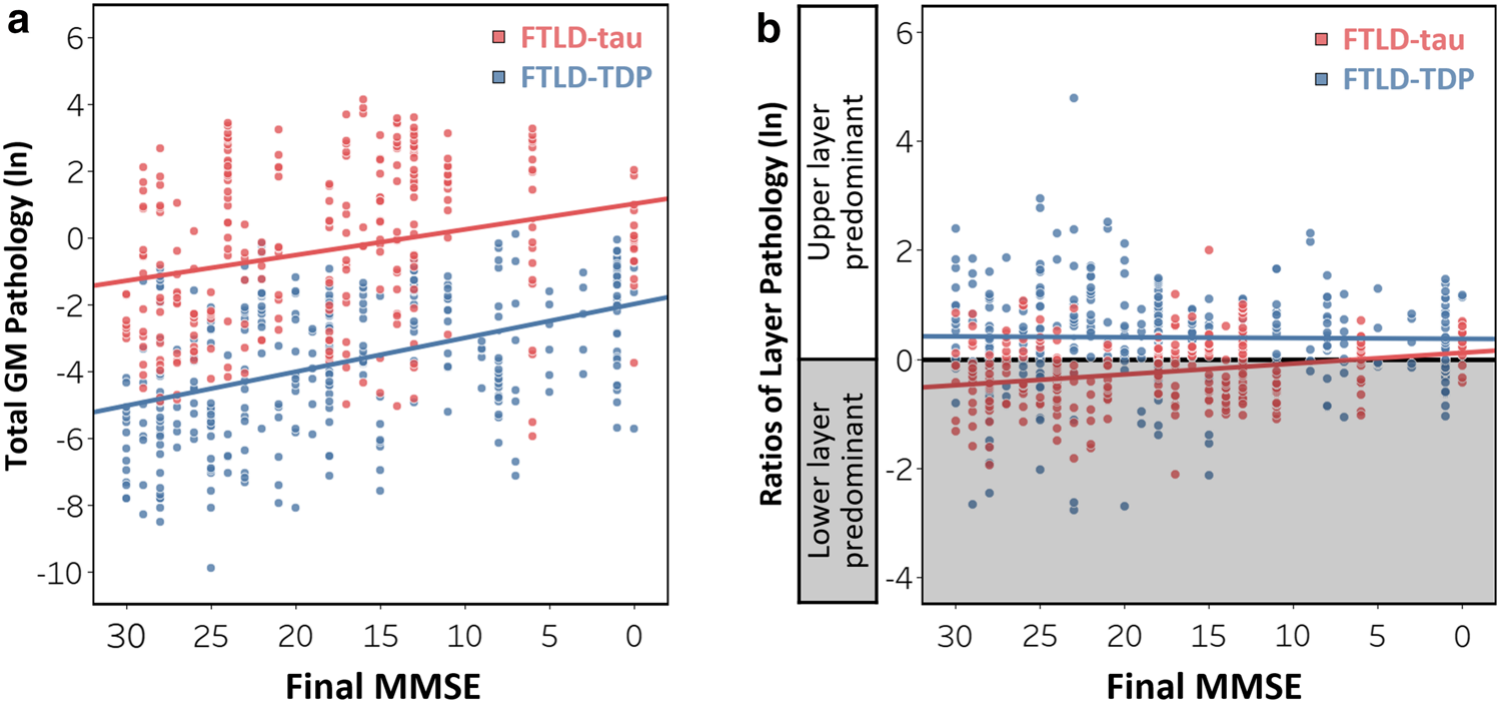
Relationships between laminar distributions of pathology and global cognitive impairment in FTLD-tau and FTLD-TDP. **(a)** Lower MMSE score was associated with greater total gray matter (GM) pathology in both FTLD-tau (*β* = -0.08, SE = 0.02, *p* = 0.003) and FTLD-TDP (*β* = -0.10, SE = 0.02, *p* < 0.001). Lines represent the predicted total GM pathology as a function of final MMSE controlling for covariates in each model (i.e., hemisphere, region, sex, education, age at death, and time interval between final MMSE and death). (**b)** Lower MMSE score was associated with higher ratios of layer pathology in FTLD-tau (*β* = -0.02, SE = 0.01, *p* = 0.018). In contrast, there was no relationship between MMSE score and the ratio of layer pathology in FTLD-TDP (*β* = 0.001, SE = 0.01, *p* = 0.838). Lines represent the predicted ratios of layer pathology as a function of final MMSE controlling for covariates in each model (i.e., hemisphere, region, sex, education, age at death, and time interval between final MMSE and death). Scatterplots represent ratios of layer pathology per region of each patient

We explored the relationships between laminar pathology and global cognitive impairment in FTLD patients evaluated closest to death (*n* = 96). First, we found that lower MMSE scores were associated with greater total GM pathology in FTLD-tau (*β* = − 0.08, SE = 0.02, *p* = 0.003); all other effects were non-significant effects (*p* > 0.1) except region (*p* < 0.001) and age at death (*p* = 0.002). Similarly, lower MMSE scores were associated with greater total GM pathology in FTLD-TDP (*β* = − 0.10, SE = 0.02, *p* < 0.001); all other effects were non-significant (*p* > 0.1) except region (*p* < 0.001) (Fig. 7a). Despite these relationships, we found no relationship between MMSE score and the upper layer-predominant pathology characteristic of FTLD-TDP (*β* = 0.001, SE = 0.01, *p* = 0.838). In contrast, lower MMSE score was associated with higher ratios of layer pathology in FTLD-tau (*β* = − 0.02, SE = 0.01, *p* = 0.018) (Fig. 7b); all other effects were non-significant (*p* > 0.05) except region (*p* = 0.001). Next, we performed a focused subanalysis in bvFTD (*n* = 44) to avoid potential phenotypic differences in cognitive testing [73]. Similar to total FTLD groups, we found no relationship between the ratio of layer pathology and MMSE score in bvFTD-TDP (*β* = 0.003, SE = 0.01, *p* = 0.752), whereas lower MMSE score was associated with higher ratios of layer pathology in bvFTD-tau (*β* = − 0.04, SE = 0.01, *p* = 0.004). In a subset of our total FTLD cohort with behavioral changes measured close to death by the NPI-Q, we found that greater upper layer-predominant tau pathology was related to more behavioral disturbances in the total FTLD-tau group, but not the total FTLD-TDP group (Supplementary Fig. 6, online resource). These findings from the NPI-Q, in combination with MMSE data, provide converging evidence that a disproportionate accumulation of pathology in upper layers may contribute to cognitive and behavioral deficits in clinically similar FTLD-tau and FTLD-TDP patients.

### FTLD-tau and FTLD-TDP display signature laminar distributions of pathology in bvFTD and PPA

**Fig. 8:**
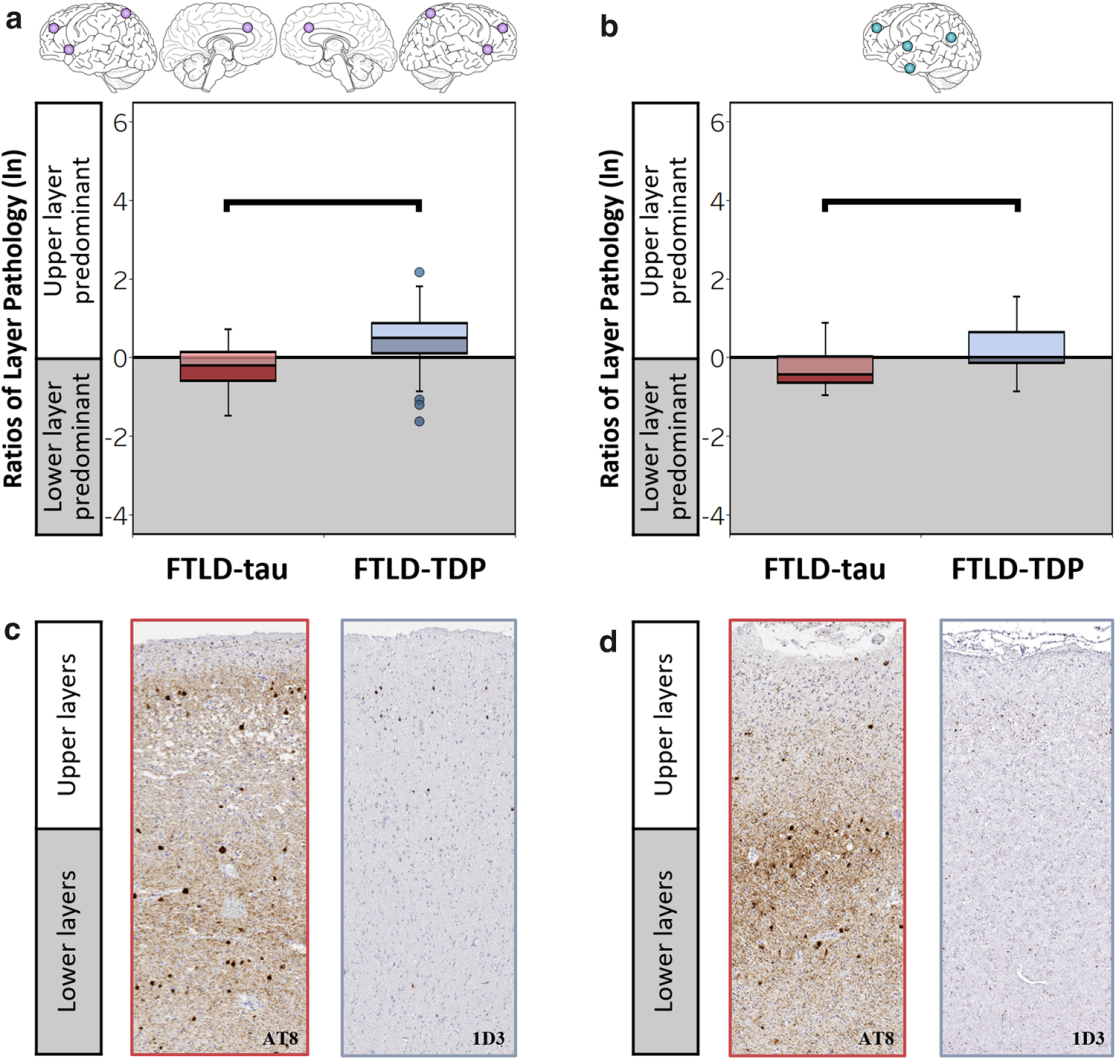
FTLD-tau and FTLD-TDP display signature laminar distributions of pathology in bvFTD and PPA. **(a)** Ratios of layer pathology in bilateral regions clinically relevant to bvFTD (i.e., aCING, aINS, MFC, pSPC). Bilateral salience/executive-related regions displayed lower ratios of layer pathology in bvFTD-tau compared to bvFTD-TDP (*β* = -0.63, SE = 0.12, *p* < 0.001). **(b)** Ratios of layer pathology in left hemisphere regions clinically relevant to PPA (i.e., MFC, aITC, SMTC, pIPC). Left language-related regions displayed lower ratios of layer pathology in PPA-tau compared to PPA-TDP (*β* = -0.49, SE = 0.09, *p* < 0.001). (**c)** Representative images of lower layer-predominant AT8-immunoreactive tau pathology (left) and upper layer-predominant 1D3-immunoreactive TDP-43 pathology (right) in the aCING region of bvFTD patients with either PiD (left) or TDP-A (right). (**d)** Representative images of lower layer-predominant AT8-immunoreactive tau pathology (left) and upper-predominant 1D3-immunoreactive TDP-43 pathology (right) in the left SMTC region of PPA patients with either PiD (left) or TDP-A (right)

We tested whether distinct clinical syndromes and regions clinically relevant to these syndromes predict similar laminar distributions of pathology by examining our largest dementia subgroups, bvFTD and PPA. Consistent with total group results, we first found lower ratios of layer pathology in FTLD-tau compared to FTLD-TDP in both bvFTD (*β* = − 0.50, SE = 0.09, *p* < 0.001) and PPA (*β* = − 0.73, SE = 0.14, *p* < 0.001) with a significant effect of region in each model (*p* < 0.04). Next, we repeated these analyses in regions clinically relevant to bvFTD and PPA. In bilateral salience/executive-related regions implicated in bvFTD, we found a lower ratio of layer pathology in bvFTD-tau compared to bvFTD-TDP (*β* = − 0.63, SE = 0.12, *p* < 0.001) (Fig. 8a); all other effects were non-significant (*p* > 0.2). Similarly, in left language regions underlying PPA, we found a lower ratio of layer pathology in PPA-tau compared to PPA-TDP (*β* = − 0.46, SE = 0.21, *p* = 0.046) (Fig. 8b); all other effects were non-significant (*p* > 0.4).

## Discussion

This comparative study finds that FTLD tauopathies feature relatively greater tau burden in lower layers and WM, while TDP-43 proteinopathies display relatively greater TDP-43 burden in upper layers with minimal WM involvement. Distinct laminar distributions of pathology were specific to proteinopathy and largely consistent across the isocortex and FTLD subgroups, providing new evidence that clinically relevant regions and clinically distinct subgroups are not significant predictors of laminar changes. These data support the hypothesis that prominent lower layer changes help distinguish FTLD-tau from FTLD-TDP. As a result, tau and TDP-43 may not affect the same cells and neural circuits that interconnect regions, despite the evidence for similar large-scale dysfunction/degeneration of clinically relevant networks in FTD-FTLD patients. Therefore, pathologic changes to layer-specific neurons and their predictable feedforward/feedback projections may inform models of neurodegenerative disease and guide the development of biomarkers sensitive to laminar pathologic change.

AD-type neurofibrillary tangles accumulate in mostly cortical layers III and V of clinical AD patients, forming a well-documented bilaminar distribution [11, 16, 54, 75]. However, laminar changes in FTD patients are far less established. Previous examinations have focused on only select inclusions in small cohorts of neuropathologically defined FTLD with limited, if any, clinical comparisons. By accounting for demographic factors, regions sampled, and subgroup classifications, our large comparative study of digitally measured pathologic burden addresses the limitations of prior studies to advance our understanding of the unique aggregation of FTLD pathologies in heterogeneous FTD syndromes. Despite known pathologic subtypes and distinct clinical phenotypes, we find that lower layer-predominant tau pathology is a common feature of FTLD-tau and upper layer-predominant TDP-43 pathology is a unifying pattern in FTLD-TDP (Fig. 2–4). Graphical representations of pathology appeared bilaminar in select subgroups (Fig. 4), but analyses comparing PiD, ALS, and total FTLD groups suggest that PiD and ALS still have distinct patterns (data not shown). In PiD, tau burden may appear bilaminar due to frequent Pick body accumulation in upper layers and prominent Pick cells in lower layers [5, 38, 43]. In ALS and TDP subtypes B-E, multiple types of TDP-43 inclusions are known to accumulate in both upper and lower layers, contributing to subtype classification criteria [19, 20, 53, 56, 58]. However, our layer ratios of total pathologic burden likely capture threads and other small pathologies incompletely assessed by previous studies, producing novel results that indicate that TDP-43 burden is distinctly greater in upper layers even when infrequent lower layer inclusions are present in various FTLD-TDP subtypes. In contrast, tau burden has a predilection for lower layers, regardless of upper layer involvement or type of tauopathy.

Neurodegenerative diseases such as AD often co-occur with FTLD [83], and select studies of PiD, CBD, PSP, and ALS with concomitant AD have found that AD-type tangles accumulate more often in supragranular layers II–III as opposed to infragranular layers V–VI [5, 34, 37–39]. Co-pathologies were relatively infrequent in our FTLD cohorts, but when present, were mostly restricted to regions outside the isocortex including limbic areas, subcortex, and brainstem (Table 1). To address the possibility that laminar distributions of FTLD pathology are altered by co-morbid pathology, we excluded FTLD brains with known co-morbid pathology, including low-to-high ADNC. In the remaining “pure” FTLD brains, we replicated our main findings of lower layer-predominant pathology in FTLD-tau and upper layer-predominant pathology in FTLD-TDP. While we cannot rule out a contribution of mixed pathology on laminar patterns in FTLD, co-morbid pathology appeared to have little effect on differences observed between FTLD-tau and FTLD-TDP.

Our large regional investigation teased apart the potential effects of regional variations in microstructure and function on laminar pathology. We demonstrated that unique laminar distributions of pathology associated with FTLD-tau and FTLD-TDP were largely consistent across regions with specialized functions (i.e., idiotypic, association, and paralimbic association regions) and intrinsic variations in laminar cytoarchitecture (e.g., agranular versus granular cortex) (Fig. 4). Region was often a significant predictor of laminar pathology in our between-group models, suggesting that we cannot entirely rule out region-specific contributions to laminar profiles in FTLD. Future investigations of the cells occupying each cortical layer will help determine which layer-specific cell populations are most vulnerable to degeneration among these diverse but interconnected regions in FTLD.

Pathologic proteins including FTLD-type tau and TDP-43 may seed and propagate via synaptically connected brain regions [25, 30, 52, 78]. Postmortem human tissue cannot directly track the temporal sequence of cell-to-cell transmission, but known laminar circuits represent a neuroanatomical model useful for testing predictions made by transneuronal spread hypotheses [26]. Specifically, distinct laminar profiles of FTLD pathology may indirectly point to which layer-specific neurons are influencing spread of pathology into connected cortices and into juxtacortical WM comprised of both efferents exiting from and afferents arriving to cortical layers. Accordingly, the current study compared laminar pathology to neighboring WM and between interconnected regions with varied disease severity. We classified regional disease severity using total GM pathologic burden and histopathologic staging data in FTLD [18–20, 43, 50] (Supplementary Tables 2–3, online resource), permitting the comparison of laminar distributions of pathology between regions with mild pathology (later involved) and severe pathology (earlier involved). In FTLD-tau, we found that severe pathology/earlier involved regions displayed greater tau pathology in lower layers and WM compared to mild pathology/later involved regions (Fig. 6, Supplementary Fig. 3, online resource). Furthermore, greater WM tau pathology was related to relatively greater lower layer tau pathology (Fig. 6c). One interpretation of these results is that select regions are vulnerable to early and preferential tau accumulation in lower layers and WM before tau spreads into later involved regions with bilaminar susceptibilities to tau deposits. Alternatively, initial tau distributions may be bilaminar, but there is selective pathologic accumulation in and progressive degeneration of lower layers, especially in the earliest involved regions that accrue the most severe pathologic burden. Indeed, a previous study of CBD showed that earlier onset and shorter disease duration were associated with bimodal/bilaminar distributions of tau pathology [7]. While we cannot rule out the unique contributions of high pathologic burden in WM in select pathologic subgroups such as CBD, pathologic subgroup was not a significant predictor in models that adjusted for subgroups (data not shown). Taken together, tau pathology may have a predilection for WM fibers due in part to layer-specific neurons (especially infragranular neurons) disproportionately targeted by tau pathology.

Pathologic spread of TDP-43 pathology may not follow the same laminar pathways as tau pathology according to our regional analyses in FTLD-TDP. Specifically, we found that laminar distributions of pathology were not associated with WM pathology and TDP-43 remained upper layer-predominant between regions of different pathologic severity or stage of involvement (Fig. 6, Supplementary Fig. 2, online resource). Consistent with a previous study in FTLD-TDP [9], these data may reflect the propensity of TDP-43 proteinopathies to accumulate and spread among upper layers compared to lower layers and WM. However, more bilaminar distributions of TDP-43 pathology have been shown to be related to increasing disease duration [9], a pattern we did not observe possibly due in part to contrasting methodologic designs. Indeed, our current study measured all immunoreactive inclusions in carefully delineated layers in a large diverse FTLD-TDP cohort as opposed to the previous study that only quantified a subset of TDP-43 inclusions without delineating cortical layers in a smaller FTLD-TDP cohort. The anatomical pathways by which pathology can spread are many, but the regional laminar patterns of pathology observed here provide preliminary evidence that FTLD-tau and FTLD-TDP may spread via different neural circuits. Upper layer-predominant TDP-43 pathology suggests corticocortical circuits may be the primary mode of transmission between cortices throughout disease progression in FTLD-TDP. In contrast, widespread interlaminar tau suggests that more diverse circuits mediate tau spread in FTLD-tau, including infragranular pathways that uniquely project to both cortical and subcortical regions.

The histopathologic correlates of cognitive decline remain elusive in much of the FTLD spectrum. To address this, we compared laminar pathology to performance on the MMSE closest to death, a neuropsychological exam that measured the severity of global cognitive impairment in the majority of our cohort. First, we found greater pathology in total GM was related to greater cognitive impairment in both FTLD groups (Fig. 7a), which validates our digital measurements and supports previous work that suggests tau and TDP-43 aggregation are associated with dementia severity [19, 20, 43, 50, 79]. Second, we found a novel relationship in FTLD-tau such that greater cognitive impairment was associated with a higher ratio of layer pathology corresponding to bilaminar/upper layer-predominant tau burden (Fig. 7b). These results were replicated in our largest dementia subgroup bvFTD (*n* = 44) to account for potential confounds of clinical phenotype on testing [73]. In contrast to FTLD-tau, we found no relationship between the ratio of layer pathology and MMSE performance in total FTLD-TDP or bvFTD-TDP. Interestingly, we found similar results in both FTLD-tau and FTLD-TDP when we compared laminar pathology to the severity of behavioral disturbances as measured by the NPI-Q [23, 46] (Supplementary Fig. 6, online resource). One explanation for these findings is that global cognitive and behavioral impairment may be closer related to upper layer degeneration than lower layer degeneration, regardless of the underlying FTLD proteinopathy. Thus, a common feature of clinically similar FTLD-tau and FTLD-TDP patients (e.g., bvFTD) may be the degeneration of supragranular corticocortical circuits that connect numerous higher association cortices [84]. Indeed, a common clinical impairment in bvFTD is disinhibition, a behavior recently linked to aberrant neural activity potentially mediated by supragranular circuits in the frontal cortex of bvFTD patients [41]. We had limited harmonized clinical data near end stage of disease for our clinicopathologic analyses. While we carefully accounted for time interval between clinical testing and autopsy in our models and restricted analyses to data collected close to death, shorter time intervals are an inherent challenge to retrospective clinicopathologic studies. However, these data are important as there is currently no neuroimaging modality capable of diagnosing FTLD neuropathology during life (especially at the resolution of cortical layers), necessitating our present postmortem investigation. Therefore, while two global clinical scales (MMSE and NPI-Q) produced converging evidence that laminar distributions of pathology may influence clinical features of FTLD patients, these findings remain exploratory. Further evaluation is needed in future investigations with more detailed and consistent longitudinal assessments of cognition and behavior as they become available.

Our comparative study included a diverse range of clinical phenotypes to determine whether laminar pathology is related to FTD presentations underrepresented or missed in previous laminar studies. Of note was the inclusion of bvFTD and PPA, our two largest clinical dementia subgroups that have a similar prevalence of underlying tauopathies or TDP-43 proteinopathies. We show for the first time that regardless if patients presented with bvFTD or PPA, tauopathies had a common lower layer-predominant profile of pathology whereas TDP-43 proteinopathies had a characteristic upper layer-predominant profile of pathology. It is important to note that laminar distributions of tau and TDP-43 pathology remained distinguishable even when our analyses were restricted to regions clinically relevant to bvFTD (bilateral salience/executive-related regions) [76, 87, 89, 91, 92, 104] and PPA (left language-related regions) [64, 85, 86] (Fig. 8). Due to the rarity of the semantic variant of PPA with FTLD-tau and the non-fluent variant of PPA with FTLD-TDP [27, 65, 94], we were unable to test for differences between these subgroups in our current cohort. Divergent laminar distributions of FTLD pathology in bvFTD or PPA hold biological and clinical significance given the anatomy of cortical layer connectivity. Namely, supragranular layers are enriched for neurons with corticocortical connections, while lower layers have a mix of neurons that connect a range of cortical, subcortical, brainstem, and thalamic structures [14, 84, 93]. These neural circuits all make diverse connections with larger cognitive networks implicated in FTD syndromes in ways not well understood. However, it is conceivable that pathologic changes to separate cortical layers and regions may converge onto a common network downstream that leads to clinically indistinguishable presentations [21, 101, 104]. Given the proteinopathy-specific distributions of pathology across cortical layers observed here, selective laminar neurodegeneration may influence the clinical profile via distinct anatomical pathways. Thus, pathologic changes to select cortical layers may inform the neural substrates of cognition and behavior in FTD, and if detected during life, could improve diagnoses and guide new disease-modifying approaches for FTLD patients.

The present study included nine FTLD proteinopathies and five clinical dementia or motor syndromes to advance our understanding of laminar pathology in the heterogeneous populations of FTLD. We calculated ratios of layer pathology to preserve the relative distribution within a region and individual patient and to permit the comparison of disparate proteinopathies independent of variability in overall burden. However, this investigation was not without some limitations. Some of our pathologic and clinical subgroups were relatively small due in part to the rarity of FTLD, requiring future studies to include multicenter participation to replicate and generalize our findings. While we sampled up to ten different anatomical regions, there were missing data for some regions due to varied tissue integrity, exhausted tissue, or evolution of brain sampling from our brain bank over the past 35 years. Linear mixed-effects models accounted for missing data, but more complete regional sampling will be performed in prospective autopsies. Given that the current study sampled one section per region available, more expansive sampling of regions using a larger series of tissue or thicker sections will be important to future laminar studies. Lastly, new examinations of laminar neurodegeneration will further elucidate the laminar patterns of disease in FTLD as reliable methods are developed and validated.

In conclusion, our results suggest that laminar distributions of pathology are relatively specific to proteinopathies, with an upper layer-predominant profile in FTLD-TDP compared to a lower layer-predominant profile in FTLD-tau. Furthermore, we find that patients with the same bvFTD or PPA clinical syndromes still display distinct laminar profiles of tau and TDP-43 pathology, even within regions that contribute to the network dysfunction underlying the syndromes. Therefore, macroscopic abnormalities in networks may have dissimilar microscopic or mesoscopic underpinnings based on the involvement of select cortical layers differentially targeted by FTLD-tau and FTLD-TDP. Given the predictable connections to and from cortical layers, it follows that specific feedforward and feedback circuits may contribute to patterns of propagation, neurodegeneration, and clinical presentations in ways not currently understood in FTD-FTLD patients. Microscopic analyses of cortical layers provide an advantageous anatomical framework for mapping and understanding the local cellular effects of FTLD proteinopathy on macroscopic network degeneration and clinical symptomology [76, 77, 101]. Moreover, laminar determinants of clinical features in FTD may inform antemortem biomarkers of proteinopathy, especially with the use of high-resolution MRI measures of cortical layers and connectivity under development [12, 51, 96, 100].

## Supplementary Information

Below is the link to the electronic supplementary material.

Supplementary file1 (DOCX 6792 kb)

## Data Availability

The datasets collected and/or analyzed during the current study are available from the corresponding author on reasonable request.
