# Supplementary material for: Signature laminar distributions of pathology in frontotemporal lobar degeneration
Source: Acta Neuropathol. 2022 Jan 8;143(3):363–82. doi: 10.1007/s00401-021-02402-3 (PMC8858288; doi:10.1007/s00401-021-02402-3)
Supplement: Supplementary file 1 — Supplementary file1 (DOCX 6792 kb) [file 401_2021_2402_MOESM1_ESM.docx]

**SUPPLEMENTARY MATERIAL**

| **a** | **Total FTLD groups** | |  |  |  | | | | |  | |  | |  |  |
| --- | --- | --- | --- | --- | --- | --- | --- | --- | --- | --- | --- | --- | --- | --- | --- |
|  |  |  | **Total**  **patients** | **Hemi** | **M1** | **aCING** | **aINS** | **aOFC** | **MFC** | **aITC** | **SMTC** | **pIPC** | **pSPC** | **V1** | *Total  sections* |
|  | **FTLD-tau** | | 73 | Left | 38 | 28 | 11 | 31 | 39 | 9 | 32 | 31 | 10 | 42 | *271* |
|  |  |  |  | Right | 28 | 23 | 8 | 30 | 31 | 8 | 23 | 27 | 11 | 31 | *220* |
|  |  | |  | *Total* | *66* | *51* | *19* | *61* | *70* | *17* | *55* | *58* | *21* | *73* | *491* |
|  | **FTLD-TDP** | | 97 | Left | 40 | 38 | 6 | 47 | 44 | 6 | 38 | 36 | 8 | 39 | *302* |
|  |  |  |  | Right | 44 | 36 | 9 | 39 | 34 | 7 | 33 | 35 | 8 | 45 | *290* |
|  |  |  |  | *Total* | *84* | *74* | *15* | *86* | *78* | *13* | *71* | *71* | *16* | *84* | *1083* |
|  |  | |  |  |  | | | | |  | |  | |  |  |
| **b** | **Pathologic subgroups** | |  |  |  | | | | |  | |  | |  |  |
|  |  |  | **Total**  **patients** |  | **M1** | **aCING** | **aINS** | **aOFC** | **MFC** | **aITC** | **SMTC** | **pIPC** | **pSPC** | **V1** | *Total  sections* |
|  | **FTLD-tau** | ***CBD*** | 14 |  | 10 | 9 | 9 | 15 | 16 | 7 | 9 | 9 | 9 | 14 | *107* |
|  |  | ***PiD*** | 14 |  | 14 | 10 | 3 | 9 | 13 | 2 | 10 | 14 | 2 | 16 | *93* |
|  |  | ***PSP*** | 36 |  | 34 | 26 | 6 | 34 | 34 | 6 | 31 | 29 | 8 | 36 | *244* |
|  |  | ***TauU*** | 9 |  | 8 | 6 | 1 | 3 | 7 | 2 | 5 | 6 | 2 | 7 | *47* |
|  | **FTLD-TDP** | ***TDP-A*** | 25 |  | 20 | 20 | 7 | 24 | 22 | 4 | 18 | 18 | 6 | 21 | *160* |
|  |  | ***TDP-B*** | 17 |  | 17 | 14 | 3 | 15 | 12 | 2 | 11 | 12 | 4 | 16 | *106* |
|  |  | ***TDP-C*** | 16 |  | 14 | 15 | 2 | 17 | 17 | 3 | 12 | 15 | 2 | 11 | *108* |
|  |  | ***TDP-E*** | 5 |  | 3 | 3 | 2 | 4 | 3 | 2 | 4 | 3 | 2 | 5 | *31* |
|  |  | **ALS** | 34 |  | 30 | 22 | 1 | 26 | 24 | 2 | 26 | 23 | 2 | 31 | *187* |
|  |  | |  |  |  | | | | |  | |  | |  |  |
| **c** | **Clinical subgroups** | |  |  |  | | | | |  | |  | |  |  |
|  |  |  | **Total**  **patients** |  | **M1** | **aCING** | **aINS** | **aOFC** | **MFC** | **aITC** | **SMTC** | **pIPC** | **pSPC** | **V1** | *Total  sections* |
|  | **FTLD-tau** | ***bvFTD*** | 28 |  | 25 | 20 | 8 | 24 | 15 | 5 | 15 | 24 | 11 | 27 | *174* |
|  |  | ***CBS*** | 5 |  | 5 | 4 | 0 | 5 | 5 | 0 | 5 | 5 | 0 | 4 | *33* |
|  |  | ***PPA*** | 16 |  | 15 | 8 | 9 | 21 | 18 | 10 | 16 | 8 | 8 | 17 | *130* |
|  |  | ***PSPS*** | 24 |  | 21 | 19 | 2 | 21 | 22 | 2 | 19 | 21 | 2 | 25 | *154* |
|  | **FTLD-TDP** | ***bvFTD*** | 50 |  | 44 | 44 | 14 | 40 | 52 | 10 | 40 | 39 | 14 | 45 | *342* |
|  |  | ***CBS*** | 2 |  | 1 | 1 | 0 | 2 | 1 | 0 | 1 | 1 | 0 | 1 | *8* |
|  |  | ***PPA*** | 17 |  | 15 | 9 | 0 | 14 | 11 | 1 | 7 | 10 | 0 | 13 | *80* |
|  |  | ***ALS*** | 28 |  | 24 | 20 | 1 | 21 | 23 | 2 | 23 | 21 | 2 | 25 | *162* |
|  |  |  |  |  |  |  |  |  |  |  |  |  |  |  |  |

**Supplementary Table 1: Isocortical regions available for laminar analyses in pathologic and clinical subgroups of each FTLD proteinopathy.**

A total of 1,083 tissue sections met inclusion criteria for laminar analyses in the current study. One section per region was examined in both hemispheres where available. Sections available per hemisphere are combined in each region for clarity in pathologic subgroups (**b**) and clinical subgroups (**c**). We excluded 341 tissue sections due to artifacts or tears that prevented the examination of cytoarchitecture and segmentation of all cortical laminae. Primary motor cortex (M1), anterior cingulate cortex (aCING), anterior insular cortex (aINS), middle frontal cortex (MFC), orbital frontal cortex (OFC), anterior inferior temporal cortex (aITC), superior/middle temporal cortex (SMTC), posterior inferior parietal cortex (pIPC), posterior superior parietal cortex (pSPC), and primary visual cortex (V1), frontotemporal lobar degeneration (FTLD), transactive response DNA-binding protein of 43 kDa (TDP), Pick’s disease (PiD), corticobasal degeneration (CBD), progressive supranuclear palsy (PSP), unclassifiable tauopathy (TauU), transactive response DNA-binding protein of 43 kDa (TDP), amyotrophic lateral sclerosis (ALS).

| **Isocortical Region** | **Stage of involvement by pathologic subgroups of FTLD proteinopathies** | | | | | | | |
| --- | --- | --- | --- | --- | --- | --- | --- | --- |
|  | **PiD** | | **PSP** | | **TDP-A, -B, -C** | | **ALS** | |
|  | **Phase** | **Stage** | **Step** | **Stage** | **Pattern** | **Stage** | **Stage** | **Stage** |
| **M1** | 3 | Later | 3/4 | Earlier | 3 | Later | 1 | Earlier |
| **aCING** | 1a | Earlier | X | X | 2 | Earlier | X | X |
| **aINS** | 1a | Earlier | X | X | 2 | Earlier | X | X |
| **MFC** | 1b | Earlier | 3/4 | Earlier | 2 | Earlier | 2 | Earlier |
| **aOFC** | 1b | Earlier | X | X | 1 | Earlier | 3 | Later |
| **aITC** | X | X | X | X | X | X | X | X |
| **SMTC** | 1b | Earlier | 4/5 | Later | 2 | Earlier | 3 | Later |
| **pIPC** | 1b | Earlier | 4/5 | Earlier | 3 | Later | X | X |
| **pSPC** | X | X | X | X | X | X | X | X |
| **V1** | 4 | Later | 5/6 | Later | 4 | Later | 3 | Later |

**Supplementary Table 2: Isocortical regions categorized into earlier or later stage of involvement.**

Previous investigations of select tauopathies (i.e., PiD, PSP) and TDP-43 proteinopathies (i.e., ALS, FTLD-TDP types A-C) have identified unique accumulations of tau and TDP-43 pathology across the cortex that may reflect stages of pathologic propagation between connected regions.**^18-20,41,48^** Based on these histopathologic staging data reported previously, we categorized regions of the current study into earlier vs later stages of involvement when explicitly classified. Primary motor cortex (M1), anterior cingulate cortex (aCING), anterior insular cortex (aINS), middle frontal cortex (MFC), orbital frontal cortex (OFC), anterior inferior temporal cortex (aITC), superior/middle temporal cortex (SMTC), posterior inferior parietal cortex (pIPC), posterior superior parietal cortex (pSPC), and primary visual cortex (V1), frontotemporal lobar degeneration (FTLD), Pick’s disease (PiD), progressive supranuclear palsy (PSP), transactive response DNA-binding protein of 43 kDa (TDP), amyotrophic lateral sclerosis (ALS), X = region omitted from analyses due to lack of classification previously**^18-20,41,48^**

| **Isocortical Region** | **Mild vs severe pathology in pathologic subgroups of FTLD proteinopathies** | | | | | | | | |
| --- | --- | --- | --- | --- | --- | --- | --- | --- | --- |
|  | **PiD** | **CBD** | **PSP** | **TauU** | **TDP-A** | **TDP-B** | **TDP-C** | **TDP-E** | **ALS** |
| **M1** | Mild | Mild | Severe | Mild | Severe | Severe | Severe | Severe | Severe |
| **aCING** | Severe | Severe | Severe | Mild | Severe | Severe | Severe | Severe | Mild |
| **aINS** | Mild | Severe | Severe | Severe | Severe | Severe | Severe | Severe | Severe |
| **MFC** | Severe | Severe | Severe | Severe | Mild | Severe | Mild | Severe | Mild |
| **aOFC** | Severe | Severe | Mild | Severe | Mild | Severe | Severe | Severe | Mild |
| **aITC** | Severe | Severe | Severe | Severe | Severe | Severe | Severe | Severe | Mild |
| **SMTC** | Severe | Severe | Mild | Mild | Severe | Severe | Mild | Severe | Mild |
| **pIPC** | Mild | Mild | Mild | Severe | Mild | Mild | Mild | Severe | Mild |
| **pSPC** | Mild | Mild | Severe | Severe | Mild | Mild | Severe | Mild | Mild |
| **V1** | Mild | Mild | Mild | Mild | Mild | Mild | Mild | Mild | Severe |

**Supplementary Table 3: Isocortical regions categorized into mild or severe pathology.**

Regions were categorized as ‘severe’ or ‘mild’ pathology regions if the median pathologic burden in a GM region was greater than (severe designation) or lesser than (mild designation) the median pathologic burden of all GM regions combined within each pathologic subgroup. Primary motor cortex (M1), anterior cingulate cortex (aCING), anterior insular cortex (aINS), middle frontal cortex (MFC), orbital frontal cortex (OFC), anterior inferior temporal cortex (aITC), superior/middle temporal cortex (SMTC), posterior inferior parietal cortex (pIPC), posterior superior parietal cortex (pSPC), and primary visual cortex (V1), frontotemporal lobar degeneration (FTLD), transactive response DNA-binding protein of 43 kDa (TDP), Pick’s disease (PiD), corticobasal disease (CBD), progressive supranuclear palsy (PSP), unclassifiable tauopathy (TauU), transactive response DNA-binding protein of 43 kDa (TDP), amyotrophic lateral sclerosis (ALS).

| **FTLD pathologic subgroups** | | | | | |
| --- | --- | --- | --- | --- | --- |
| *Fixed Effect* | | *Numerator df* | *Denominator df* | *F value* | *p value* |
| Intercept | | 1 | 78.869 | 1.110 | .295 |
| Region | | 9 | 424.575 | 2.378 | .012 |
| Hemisphere | | 1 | 334.823 | .063 | .801 |
| Age at death | | 1 | 78.750 | .165 | .686 |
| Disease duration | | 1 | 73.926 | .122 | .727 |
| **FTLD-tau pathologic subgroups** | | **3** | **71.013** | **1.933** | **.132** |
|  | |  |  |  |  |
| *Fixed Effect* | | *Numerator df* | *Denominator df* | *F value* | *p value* |
| Intercept | | 1 | 85.229 | 2.114 | .150 |
| Region | | 9 | 518.386 | 2.849 | .003 |
| Hemisphere | | 1 | 217.886 | .072 | .788 |
| Age at death | | 1 | 85.910 | .000 | .986 |
| Disease duration | | 1 | 70.987 | .599 | .441 |
| **FTLD-TDP pathologic subgroups** | | **4** | **72.360** | **1.335** | **.265** |
|  | |  |  |  |  |
| **FTLD genetic (familial/sporadic) subgroups** | | | | | |
| *Fixed Effect* | | *Numerator df* | *Denominator df* | *F value* | *p value* |
| Intercept | | 1 | 78.353 | 1.298 | .258 |
| Region | | 9 | 424.813 | 2.455 | .010 |
| Hemisphere | | 1 | 346.617 | .004 | .948 |
| Age at death | | 1 | 75.291 | .001 | .976 |
| Disease duration | | 1 | 73.139 | .356 | .552 |
| **FTLD-tau familial/sporadic subgroups** | | **1** | **76.452** | **.224** | **.637** |
|  | |  |  |  |  |
| *Fixed Effect* | | *Numerator df* | *Denominator df* | *F value* | *p value* |
| Intercept | | 1 | 87.836 | 1.525 | .220 |
| Region | | 9 | 519.997 | 2.885 | .002 |
| Hemisphere | | 1 | 230.569 | .051 | .821 |
| Age at death | | 1 | 85.253 | .036 | .850 |
| Disease duration | | 1 | 76.258 | .274 | .602 |
| **FTLD-TDP familial/sporadic subgroups** | | **1** | **79.976** | **1.249** | **.267** |
|  | |  |  |  |  |
| **FTLD clinical subgroups** | | | | | |
| *Fixed Effect* | | *Numerator df* | *Denominator df* | *F value* | *p value* |
| Intercept | | 1 | 76.493 | 5.033 | .028 |
| Region | | 9 | 425.504 | 2.519 | .008 |
| Hemisphere | | 1 | 333.325 | .062 | .803 |
| Age at death | | 1 | 76.026 | 1.257 | .266 |
| Disease duration | | 1 | 72.058 | .095 | .758 |
| **FTLD-tau clinical subgroups** | | **3** | **67.403** | **2.774** | **.048** |
| Bonferroni-corrected pair-wise comparisons of FTLD-tau clinical subgroups | | Mean difference | SE | df | p value |
| bvFTD | CBS | .478 | .198 | 66.753 | .112 |
|  | PPA | .275 | .129 | 66.293 | .223 |
|  | PSPS | .222 | .119 | 69.742 | .390 |
| CBS | bvFTD | -.478 | .198 | 66.753 | .112 |
|  | PPA | -.203 | .203 | 66.714 | 1.000 |
|  | PSPS | -.256 | .193 | 66.935 | 1.000 |
| PPA | bvFTD | -.275 | .129 | 66.293 | .223 |
|  | CBS | .203 | .203 | 66.714 | 1.000 |
|  | PSPS | -.053 | .128 | 67.052 | 1.000 |
| PSPS | bvFTD | -.222 | .119 | 69.742 | .390 |
|  | CBS | .256 | .193 | 66.935 | 1.000 |
|  | PPA | .053 | .128 | 67.052 | 1.000 |
|  | |  |  |  |  |
| *Fixed Effect* | | *Numerator df* | *Denominator df* | *F value* | *p value* |
| Intercept | | 1 | 90.313 | 1.218 | .273 |
| Region | | 9 | 518.374 | 2.864 | .003 |
| Hemisphere | | 1 | 230.125 | .025 | .875 |
| Age at death | | 1 | 82.553 | .010 | .921 |
| Disease duration | | 1 | 80.910 | .634 | .428 |
| **FTLD-TDP clinical subgroups** | | **3** | **107.066** | **.724** | **.540** |

**Supplementary Table 4: Pathologic, genetic, and clinical subgroup analyses in FTLD-tau and FTLD-TDP**


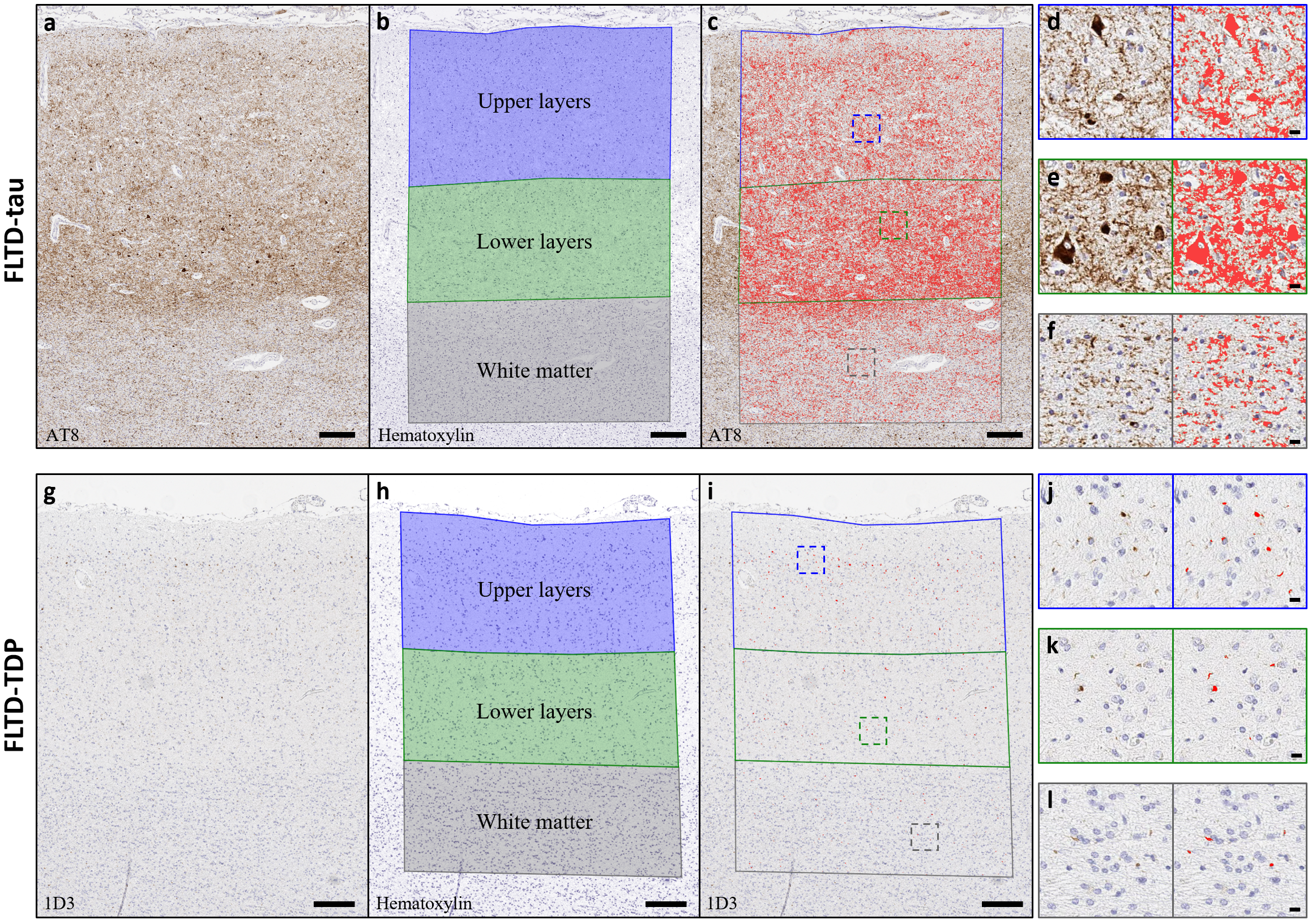
**Supplementary Fig. 1: Digital quantitation of pathology within delineated laminar and white matter subregions in FTLD-tau and FTLD-TDP.**

In every immunostained section (AT8-immunoreactive tau, **panel** **a**; 1D3-immunoreactive TDP-43, **panel** **g**), the longest stretch of relatively flat isocortex (typically in the sulcal wall of a gyrus) was located to apply the belt-transect method of sampling using QuPath software. Upper layers I-III (blue), lower layers IV-VI (green), and juxtacortical white matter subregions (gray) were manually delineated in each section while blinded to any immunoreactive inclusions by visualizing the cytoarchitecture through only the blue hematoxylin channel in QuPath (**panels** **b and h**). In QuPath, we used positive pixel classifiers to digitally quantify pathologic burden as the percent area occupied (%AO) by tau- and TDP-43-immunoreactive pixels in each laminar and white matter subregion (**red overlay; panels** **c and i**). High magnification photomicrographs visualize the close correspondence between microscopic inclusions (**left side of panels d-f and j-l**) and the red overlay (**right side of panels d-f and j-l**). Images represent sections from the MFC region with severe pathologic burden in each FTLD group. Scale bars in panels a-c and g-i = 200μm. Scale bars in panels d-f and j-l = 10μm


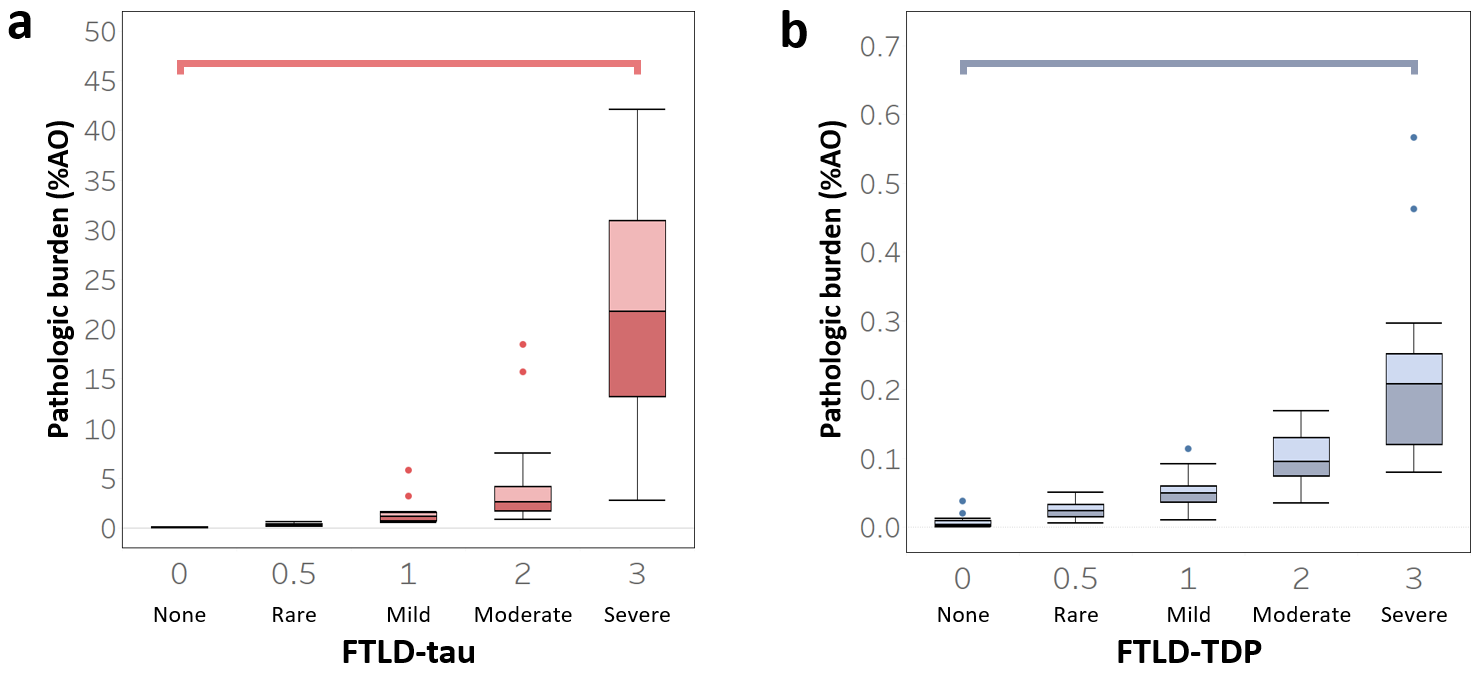
**Supplementary Fig. 2: Validation of digital quantitation of pathologic burden in FTLD-tau and FTLD-TDP.**

To validate the digitally measured pathologic burden (%AO), we completed ordinal ratings of pathologic severity using a 5-point scale in upper layers, lower layers, and white matter from all available MFC tissue of our bvFTD subgroup in each FTLD group. We found good agreement between the digitally measured pathologic burden and ordinal ratings of pathologic severity. We validated this correspondence by performing a Kruskal-Wallis analysis in each FTLD group, which determined that digital measures of pathologic burden were significantly different between each ordinal rating of tau pathology (H=49.98, p<0.001) and TDP-43 pathology (H=105.96, p<0.001). As an additional validation, we performed analyses that included the immunostaining run as a fixed effect to determine its potential contribution to our main dependent variable of interest, ratio of layer pathology. We found that the immunostaining run was not a significant predictor of ratio of layer pathology in either the FTLD-tau group (p=0.564) or the FTLD-TDP (p=0.788) group, suggesting that potential variations in immunostaining runs did not significantly influence our findings. Boxplots represent the ordinal ratings of pathologic burden of all subregions examined (i.e., upper layers, lower layers, white matter)


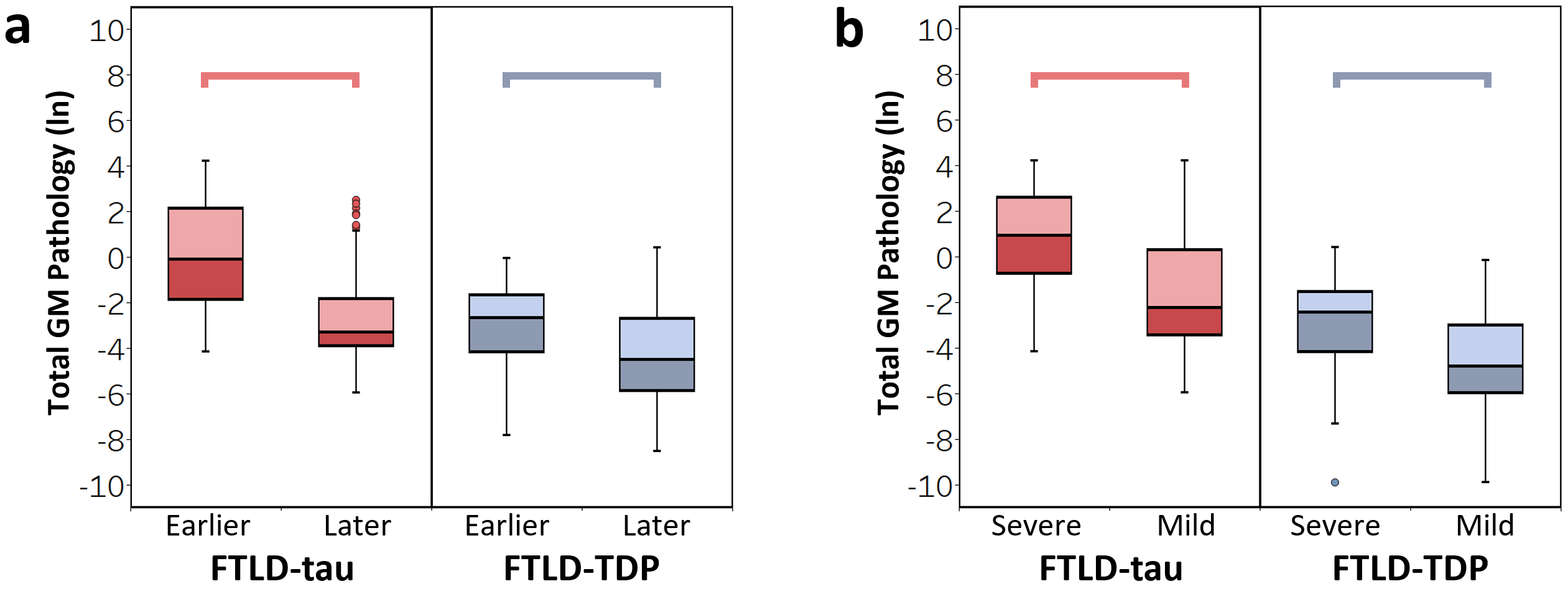
**Supplementary Fig. 3: Hypothesized disease progression between regions with high and low disease severity in FTLD-tau and FTLD-TDP.**

Hypothesized disease progression between regions was tested in each FTLD group, with the expectation that total GM pathology would be greater in earlier-involved regions and severe pathology regions compared to later-involved regions and mild pathology regions, respectively. As expected for early and later-involved regions (**Supplementary Table 2**), we determined that greater total GM pathology was found in earlier-involved regions compared to later-involved regions in each pathologic subgroup with available regional data, including PiD (β=4.38, SE=0.47, p<0.001), PSP (β=1.80, SE=0.22, p<0.001), TDP-A, -B, -C combined (β=0.80, SE=0.17, p<0.001), and ALS (β=1.30, SE=0.23, p<0.001). These results were replicated at the total group level, with earlier-involved regions displaying greater total GM pathology compared to later-involved regions in total FTLD-tau (β=1.23, SE=0.38, p=0.001) and FTLD-TDP (β=1.24, SE=0.18, p<0.001) (**Supplementary Fig. 3a**).

As expected for the complementary analysis of severe and mild pathology regions (**Supplementary Table 3**), severe pathology regions displayed greater total GM pathology compared to mild pathology regions in total FTLD-tau (β=0.97, SE=0.19, p<0.001) and FTLD-TDP (β=1.18, SE=0.14, p<0.001) (**Supplementary Fig. 3b**). These results suggest that these independent but complementary methods of categorizing regions into high or low disease severity may reflect a regional framework for disease progression by which laminar distributions of pathology can be compared. Boxplots represent ratios of layer pathology per region of each patient


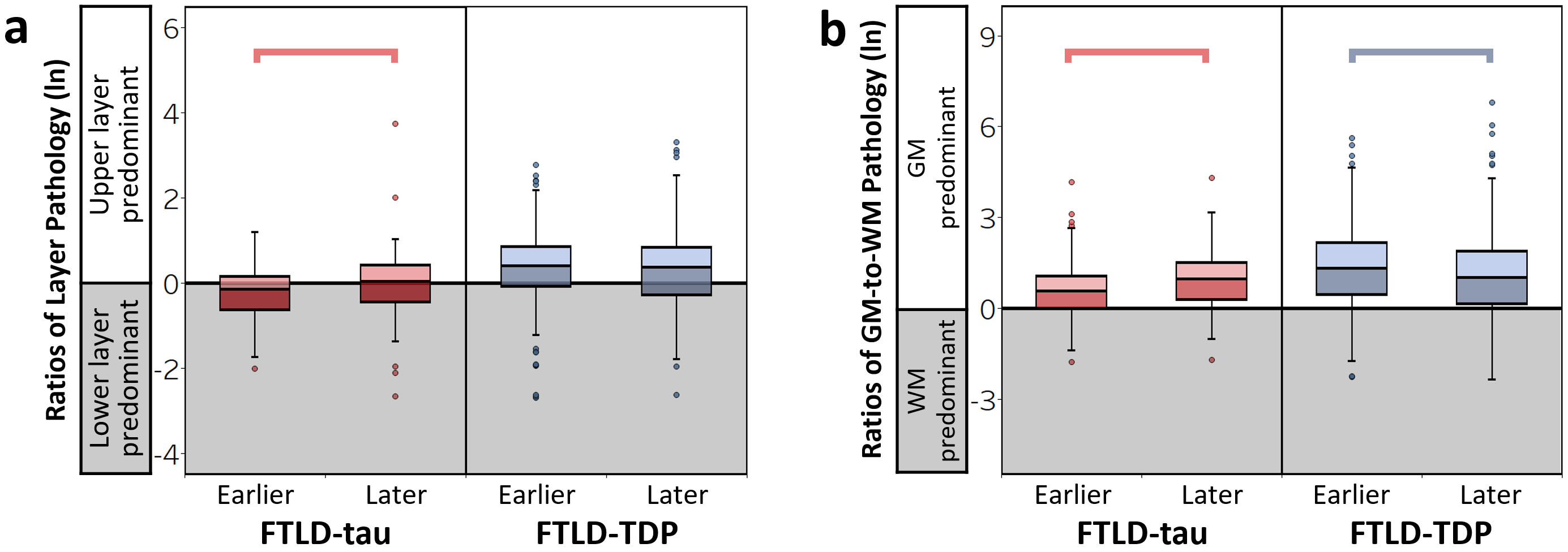
**Supplementary Fig. 4: Laminar distributions of pathology in relation to regional stage of disease and juxtacortical white matter in FTLD-tau and FTLD-TDP.**

We determined whether ratios of layer pathology or ratios of GM-to-WM pathology varied between regions involved in earlier vs later stages of disease (**Supplementary Table 2**).**^18-20,41,48^** Similar to results found in severe and mild pathology regions reported in the main results, we found lower ratios of layer pathology in earlier-involved regions compared to regions involved in later stages of pathologic disease progression in FTLD-tau (β=-0.43, SE=0.15, p=0.004). In contrast, earlier- and later-involved regions displayed similar higher ratios of layer pathology in FTLD-TDP (β=-0.09, SE=0.11, p=0.431) (**Supplementary Fig. 4a**).

Similar to above, we tested the hypothesis that the relative distribution of GM-to-WM pathology may be related to regional disease severity due to potential spread of pathology between the connected regions. In FTLD-tau, we found that earlier-involved regions displayed lower GM-to-WM ratios of tau pathology compared to later-involved regions (β=-0.60, SE=0.20, p=0.003), suggesting that tau pathology was more WM-predominant in earlier-involved regions. In FTLD-TDP, earlier-involved regions displayed higher GM-to-WM ratios of TDP-43 pathology compared to later-involved regions (β=0.46, SE=0.17, p=0.008) (**Supplementary Fig. 4b**).

We further examined potential staging of pathology (i.e., severe vs mild pathology regions) in a subset of patients in each FTLD group (33 FTLD-tau patients, 42 FTLD-TDP patients) with no missing data by using the same staging-relevant regions (MFC, aCING, SMTC, pIPC, and V1) across lobes. Consistent with our original staging analyses that included all available regions, our subanalyses with select regions sampled together find that severe pathology regions (potentially early-involved regions) display greater lower layer-predominant tau pathology compared to low pathology regions (potentially later-involved regions) in FTLD-tau only (β=-0.24, SE=0.11, p=0.029). In contrast, we find relatively high ratios of layer TDP-43 pathology were not different between severe pathology and low pathology regions in FTLD-TDP (β=0.10, SE=0.14, p=0.478). These results complement our main results and suggest that variations in region availability did not influence our consistent observation that tau pathology and TDP-43 pathology have a predilection for relatively distinct cortical layers throughout disease progression. Boxplots represent ratios of layer pathology per region of each patient


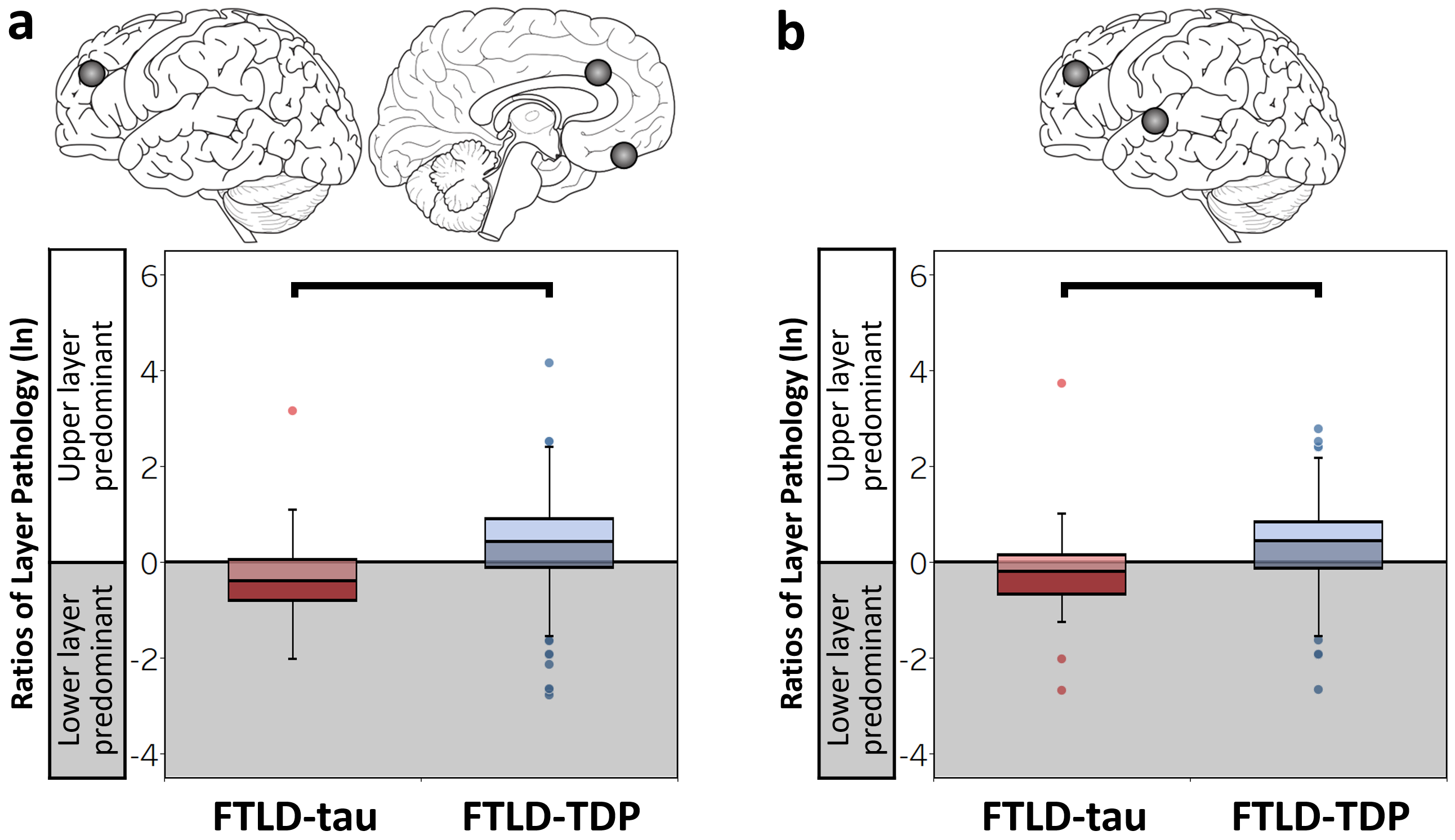
 **Supplementary Fig. 5: Laminar distributions of tau and TDP-43 pathology are distinct in patients with identical regions sampled.**

We compared laminar distributions of pathology between FTLD groups in a subset of patients with no missing data (i.e., complete data from the same regions examined). The first analysis included patients with MFC, aOFC, and aCING sampled together, representing the most frequently sampled frontal association regions in our total cohort (40 FTLD-tau, 57 FTLD-TDP). The second analysis included patients with both MFC and SMTC sampled together, representing the most frequently sampled frontotemporal association regions commonly vulnerable to both FTLD proteinopathies (47 FTLD-tau, 58 FTLD-TDP). Consistent with main analyses that include all available regions, these analyses find that FTLD-tau displays a lower layer-predominance of tau pathology, whereas FTLD-TDP displays an upper layer-predominance of TDP-43 pathology, in the model with only frontal regions (β=-0.77, SE=0.12, p<0.001) and in the model with frontotemporal regions (β=-0.60, SE=0.13, p<0.001). Boxplots represent ratios of layer pathology per region of each patient


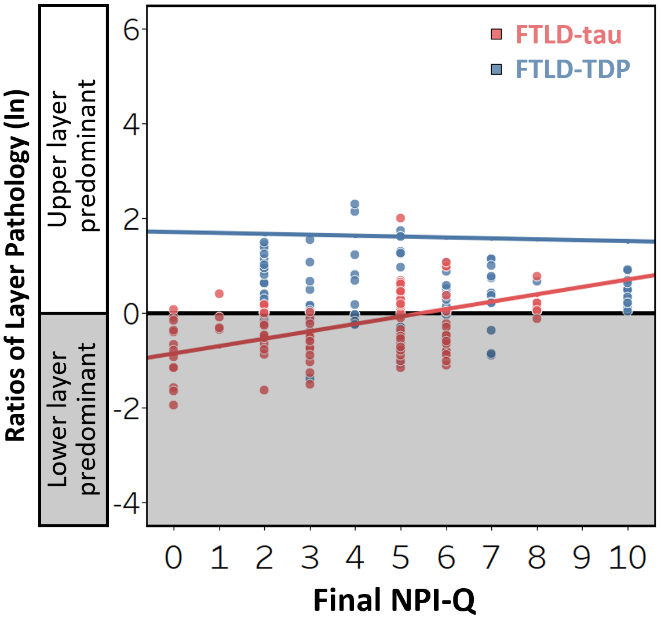
**Supplementary Fig. 6: Relationships between laminar distributions of pathology and behavioral impairment in FTLD-tau and FTLD-TDP.**

The Neuropsychiatric Inventory Questionnaire (NPI-Q) is a validated assessment of diverse behavioral changes **[23,24]** that was available within 4 years of death in a subset of 26 patients (13 FTLD-tau, 13 FTLD-TDP). Similar to our clinicopathologic analyses using MMSE scores, we find that greater upper layer-predominant tau pathology was related to more behavioral disturbances in the total FTLD-tau group (β=0.14, SE=0.03, p=0.002). In contrast, the ratio of layer TDP-43 pathology was not related to the NPI in the total FTLD-TDP group (β=-0.02, SE=0.04, p=0.663). We interpret these findings, in combination with MMSE data, as converging evidence that laminar distributions of pathology may contribute to cognitive (MMSE) and behavioral (NPI-Q) deficits in the FTD spectrum. Lines represent the predicted ratios of layer pathology as a function of final NPI-Q controlling for covariates in each model (i.e., hemisphere, region, sex, education, age at death, and time interval between final NPI-Q and death). Scatterplot represents ratios of layer pathology per region of each patient
